# Insights on forming N,O-coordinated Cu single-atom catalysts for electrochemical reduction CO_2_ to methane

**DOI:** 10.1038/s41467-020-20769-x

**Published:** 2021-01-26

**Authors:** Yanming Cai, Jiaju Fu, Yang Zhou, Yu-Chung Chang, Qianhao Min, Jun-Jie Zhu, Yuehe Lin, Wenlei Zhu

**Affiliations:** 1 State Key Laboratory of Analytical Chemistry for Life Science, School of Chemistry and Chemical Engineering, Nanjing University, Nanjing, 210023 People’s Republic of China; 2 School of Mechanical and Materials Engineering, Washington State University, Pullman, WA 99164 USA

**Keywords:** Electrocatalysis, Energy science and technology, Chemical engineering

## Abstract

Single-atom catalysts (SACs) are promising candidates to catalyze electrochemical CO_2_ reduction (ECR) due to maximized atomic utilization. However, products are usually limited to CO instead of hydrocarbons or oxygenates due to unfavorable high energy barrier for further electron transfer on synthesized single atom catalytic sites. Here we report a novel partial-carbonization strategy to modify the electronic structures of center atoms on SACs for lowering the overall endothermic energy of key intermediates. A carbon-dots-based SAC margined with unique CuN_2_O_2_ sites was synthesized for the first time. The introduction of oxygen ligands brings remarkably high Faradaic efficiency (78%) and selectivity (99% of ECR products) for electrochemical converting CO_2_ to CH_4_ with current density of 40 mA·cm^-2^ in aqueous electrolytes, surpassing most reported SACs which stop at two-electron reduction. Theoretical calculations further revealed that the high selectivity and activity on CuN_2_O_2_ active sites are due to the proper elevated CH_4_ and H_2_ energy barrier and fine-tuned electronic structure of Cu active sites.

## Abstract (web summary)

Single-atom catalysts (SACs) are promising candidates to catalyze CO_2_ reduction for the formation of high value hydrocarbons but most of the reactions yield CO. Here, the authors show a low-temperature calcining process to fabricate a carbon-dots-based SAC to efficiently convert CO_2_ to methane.

## Introduction

Electrochemical CO_2_ reduction (ECR) is becoming a viable way of both storing electric energy and removing extra CO_2_ in the atmosphere. However, unsatisfied product selectivity on cathode remains one of the major stumbling blocks present for commerciallization^1,2^. Owing to the uniform coordination structures and unique electronic properties of metal centers, single-atom catalysts (SACs) have demonstrated excellent selectivity in various catalytic reactions^3–7^. As promising candidates, SACs have been extensively explored as ECR catalysts, which boosts the product selectivity up to nearly 100%^8,9^. Though great progresses have been achieved, previous studies are generally limited to two-electron reduction products such as CO and HCOOH because of the lack of accurate manipulation in single-atom structures. For instance, traditional atomic metal–nitrogen-carbon moieties (M–N–Cs) are supported in micron-sized carbon-based substrates and form through the redispersion of aggregated metal nanoparticles under high temperature (above 800 °C)^10,11^. This process is so extreme that it causes the N- and C-containing precursors to lose the original structures and rebuild with metal atoms into the most thermodynamically preferred form, the commonly reported MN_*x*_C_4_ moiety^12,13^. In consequence, high-temperature pyrolysis makes diverse precursors to fail to tune the coordination environments which determine the intrinsic properties of obtained SACs^14^. Recently, Guan et al. reported a temperature-tuned N-coordination strategy to manipulate single-atom Cu−N_*x*_ coordination^15^. Two neighboring Cu−N_2_ sites were synthesized to form C_2_H_4_ through a C−C bond formation on two Cu atoms. But much improvement is needed in their product selectivity and Faradaic efficiency due to the ambiguous catalytic sites and the adverse Cu electronic properties when coordinated with N^16,17^. Although extensive efforts have been devoted, to our knowledge, fine tuning of SAC coordination environments to accurately change catalytic selectivity with multiple electron-reducing products remains a great challenge and are rarely investigated till now.

Conventional molecular catalysts as well-defined metal complexes possess acutely tailorable structures^18,19^, which prevail over SACs. However, they are typically involved in homogeneous catalysis^20–22^, which limits the catalytic performance by outer sphere electron transfer from the electrode in electrochemical catalysis^23,24^. To tackle this problem, researchers tried to immobilize metal phthalocyanines on carbon nanotubes to create heterogeneous catalytic surfaces^24–26^. Nevertheless, this strategy is limited to hydrophobic complexes that can resist dissolving in aqueous electrolytes. Therefore, it is of great significance to develop a more general approach to immobilize all kinds of molecular catalysts on electrodes.

**Fig. 1:**
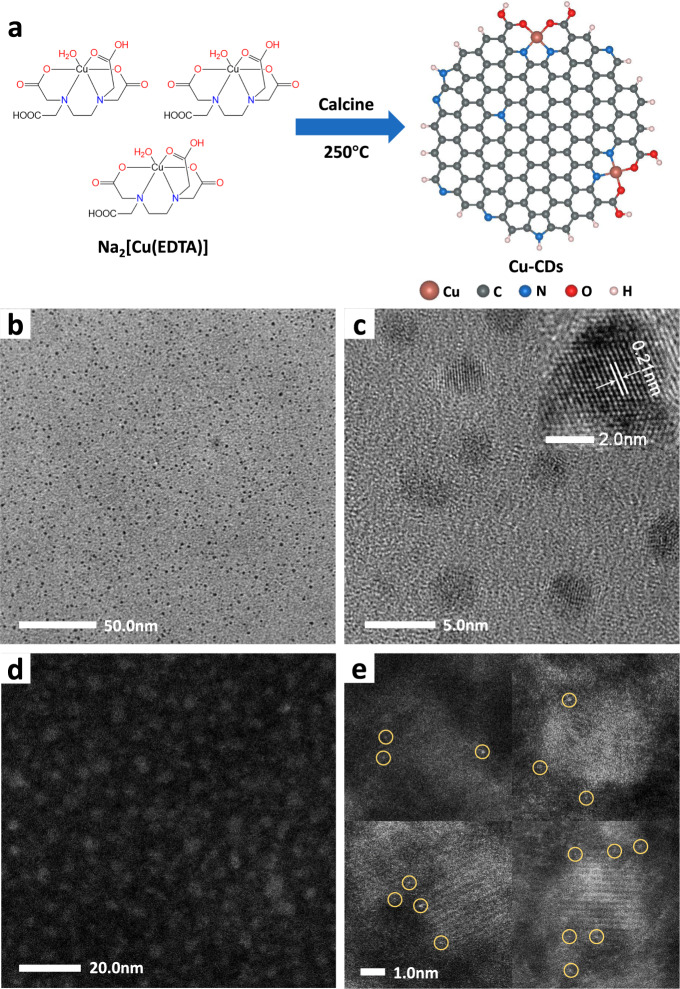
Morphology characterization of the Cu-CD catalyst. **a** Scheme of the low-temperature calcining procedure for Cu-CD catalysts. **b** Large-field of view and **c** magnified view of TEM images (the inset is the crystal lattice). **d** Relatively large-field of view and **e** typical view of HAADF-STEM images of distributed single Cu atoms in carbon dots. Yellow circles in (**e**) indicates typical single Cu atoms.

Herein, we present a carbon dots (CDs)-supported SAC prepared by a low-temperature calcining process from metal–organic complexes, which transforms the carbon containing molecular complexes into solid-state CDs while preserving the coordination environments of metal atoms. We chose copper disodium EDTA, i.e., Na_2_[Cu(EDTA)] as a demonstration (Fig. 1a). By the process of such a semi-transformed strategy, the single-atom Cu-embedded carbon dots (Cu-CDs) with coordination of two N and two O were obtained, which was the first-time introduction of N,O ligands. As a result, the catalyst exhibited the extraordinary selectivity for electrochemical reduction of CO_2_ to CH_4_ over a wide potential range from −1.14 to −1.64 V vs. RHE (reversible hydrogen electrode), as more than 99% of the CO_2_ reduction products were CH_4_. Besides, Cu-CDs presented high CH_4_ Faradaic efficiency (78%) and turn over frequency (2370 h^−1^) at −1.44 and −1.64 V, respectively. Density functional theory (DFT) calculations unveiled that the separation of CH_4_ limiting potential from other products enables exclusive producing of CH_4_. To our knowledge, this is the first report on the fabrication of SACs on functionalized CDs.

## Results

### Synthesis and structural characterization

The Cu-CDs were synthesized by calcinating Na_2_[Cu(EDTA)]·2H_2_O at 250 °C for 2 h (details in Methods), which was the minimum carbonation temperature to form CDs (Supplementary Fig. 1)^27^. The obtained Cu-CDs had a thickness between 0.7 and 1.8 nm, corresponding to 1–3 atomic layers^28^ (Supplementary Fig. 2), and transmission electron microscopes (TEM) images showed a lateral size distribution of 2–5 nm and a lattice spacing of 0.21 nm corresponding to (100) interplanar spacing^29,30^ of graphite (Fig. 1b, c and Supplementary Fig. 3). The atomic absorption spectra verified that the Cu content in Cu-CDs prepared at 250 °C was 0.44 wt%. However, when the calcination temperature increased to 300 and 350 °C, the Cu contents were decreased to 0.35 and 0.22 wt%, which indicated the destruction of –COOH functional groups so that less Cu was chelated on CDs. Meanwhile, pristine CDs without Cu-doping were also synthesized from Na_2_[H_2_(EDTA)] precursor using the identical treatment, which exhibited a similar morphology as the Cu-CDs (Supplementary Fig. 4).

The dispersive Cu atoms on Cu-CDs was confirmed by aberration-corrected high-angle annular dark-field scanning transmission electron microscopy (HAADF-STEM) (Fig. 1d, e and Supplementary Figs. 5 and 6). As shown in Fig. 1e, atom-scale bright spots represent the location of single Cu atoms that are mainly distributed around the edge of the Cu-CD framework, supporting our functional groups anchored scheme. It is extremely difficult to capture HAADF-STEM image of CDs due to the fragile intrinsic and carbon contamination under the convergent electron beam. These are the highest definition pictures we can get under boosted scanning speed but we fail to carry out electron energy loss mapping. Furthermore, the powder X-ray diffraction (XRD) spectrum (Supplementary Fig. 7) also shows no apparent peak corresponding to metallic Cu or other copper-derived crystallines on the as-synthesized Cu-CDs. Thus, the CD-supported Cu SACs were successfully synthesized. To the best of our knowledge, it was the first time that single atoms had been observed in CDs.

### Surface properties and electronic states of catalysts

**Fig. 2:**
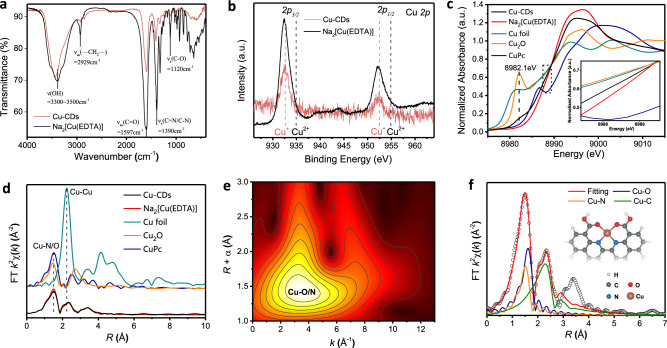
Surface properties and electronic states studies. **a** FT-IR spectra and **b** high-resolution Cu *2p* spectra of Cu-CDs and its precursor Na_2_[Cu(EDTA)]. **c** X-ray absorption near-edge structure (XANES) spectra and **d** Fourier transform (FT) EXAFS spectra at the Cu K-edge of Cu-CDs, Na_2_[Cu(EDTA)], Cu foil, Cu_2_O, and CuPc sample (inset is the magnified image of dashed box). **e** Wavelet transform (WT) of Cu-CDs. **f** EXAFS fitting curves of Cu-CDs in *R* space using backscattering paths of Cu–N, Cu–O, and Cu–C. The inset in (**f**) shows the structure of Cu sites in Cu-CDs.

To further investigate the atomic distribution and coordination environments of Cu atoms on Cu-CDs, spectral analyses were carried out. As shown in Fig. 2a, bands at ∼3434, 1597, and 1120 cm^−1^ in infrared (IR) spectrum of Cu-CDs are associated with the *ν*_O−H_, *ν*_C=O_, and *ν*_C−O_ vibrations of COOH species, and the peak at around 1390 cm^−1^ denotes the typical C=N or C–N stretching vibrations^31,32^. Compared with Na_2_[Cu(EDTA)], Cu-CDs could be verified having the functional groups coordinated with Cu ions as Na_2_[Cu(EDTA)], while the decrease in methylene antisymmetric stretching vibration at 2929 cm^-1^ indicated that the –CH_2_– species were condensed into CDs after calcination. The same results were also deduced from X-ray photoelectron spectra (XPS) (Supplementary Fig. 8). With deconvolution of the C1*s* and N1*s* peaks, the quantitative results were given in Supplementary Table 1, indicating that the surfaces of Cu-CDs were modified by abundant C–O, C=O, and N–C species. The same functional groups were detected in pristine CDs (Supplementary Figs. 9 and 10). Although Cu-CDs and CDs were similar in N content, the Cu-CDs tended to have more pyridinic N than CDs because of the oxidation by Cu^2+^ ions (Supplementary Table 2 and Supplementary Fig. 11), and pyridinic N is considered as the anchoring site for CO_2_ capture^28,33,34^.

For Cu valence state, the high-resolution Cu *2p* XPS spectra of both Cu-CDs and Na_2_[Cu(EDTA)] showed the peaks at 932.5 eV (*2p*_*3/2*_) and 952.5 eV (*2p*_*1/2*_), respectively, which are associated with the Cu^+^/Cu^0^ state (Fig. 2b) rather than the Cu^2+^ state before coordinated by EDTA^4−^. It is attributed to the strong covalent interactions between the Cu^2+^-ligand bonds^27^, causing the transfer of electrons from ligands to Cu^2+^ orbits and the decrease in Cu *2p* binding energy^35^. The fine detail of Cu-CDs at the atomic level was further investigated by synchrotron X-ray absorption spectroscopy (XAS). As shown in Fig. 2c, the absorption edge position of Cu-CDs is located between that of Cu(II) phthalocyanine (CuPc) and Cu_2_O, with a minor shift to the low-energy direction compared to Na_2_[Cu(EDTA)]. In agreement with the XPS results, the valence of Cu species in Cu-CDs is between +1 and +2, and also slightly lower than that of Na_2_[Cu(EDTA)]. For the extended X-ray absorption fine structure (EXAFS) spectra (Fig. 2d), the Cu-CD catalyst exhibited similar spectrum features to that of reference sample Na_2_[Cu(EDTA)]. They both displayed one prominent peak at 1.5 Å that corresponds to the Cu−O/N first coordination shell. The peak at around 2.3 Å is attributed to the Cu–C scattering path in higher shells^36^, and no Cu−Cu coordination peak at 2.2 Å could be detected. All of these suggested that the Cu center of Cu-CDs possesses comparable coordination environments to Na_2_[Cu(EDTA)] whose center metal is coordinated by both N and O^37,38^. The quantitative coordination configuration of Cu-CDs was further investigated by EXAFS curve fitting (Supplementary Fig. 12 and Supplementary Table 3). By comparing the fitting coordination number (CN) between Na_2_[Cu(EDTA)] (CN = 4.3) and Cu-CDs (CN = 3.6), we could determine some detachment of coordination groups in Cu-CDs. But further unveiling the respective coordination number of N and O could be a trouble because of the similar bond length of Cu–N and Cu–O, even the wavelet transform (WT) of EXAFS (Fig. 2e) failed to discriminate the N and O signals in *k*-space. Thus, three backscattering paths of N, O, and C were employed to give the best-fitting analyses (Fig. 2f and Supplementary Table 4). It confirmed that the Cu center is coordinated with two N atoms and two O atoms (Supplementary Fig. 13 and Supplementary Table 5). Therefore, the Cu atomic structure model of Cu-CDs could be deduced and shown in the inset of Fig. 2f.

### ECR performance

**Fig. 3:**
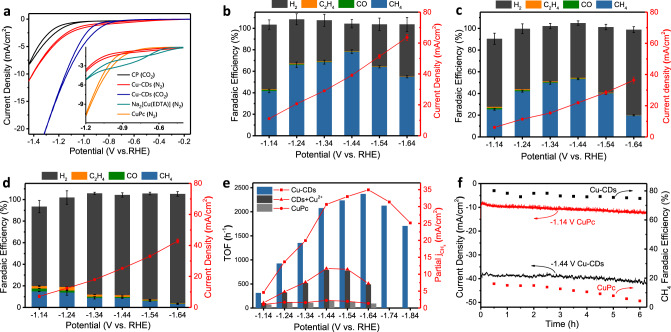
CO_2_ reduction in aqueous solution. **a** CV curves of bare carbon paper (CP), Cu-CDs, Na_2_[Cu(EDTA)], and CuPc recorded in N_2_- and CO_2_-saturated 0.5 M KHCO_3_ electrolyte with 10 mV/s scan speed. **b, c, d** Dependence of FE (left *y*-axis) and current density (based on geometric surface area, right *y*-axis) of (**b**) Cu-CDs, (**c**) CDs+Cu^2+^, and (**d**) CuPc on the applied potential. **e** Partial CH_4_ current density plots and TOFs of Cu-CDs, CDs+Cu^2+^, and CuPc at different applied potentials. **f** Stability test of Cu-CDs and CuPc at their highest ECR Faradaic efficiency potentials.

The electrocatalytic activity of the above-mentioned catalysts toward ECR were performed in an H cell employing CO_2_-saturated 0.5 M KHCO_3_ aqueous solution as electrolyte (details in Supplementary Information). As described by the cyclic voltammetry (CV) curves in Fig. 3a, there was a significant cathodic current increase recorded in Cu-CD electrode after purged with CO_2_ contrary to N_2_-saturated KHCO_3_ solution. More importantly, different from the weak and broad waves between −0.5 V and −1.2 V observed in Na_2_[Cu(EDTA)], there were no obvious Cu redox peaks in CV curves of Cu-CD and CuPc electrodes^26,39^. Taken together, these results demonstrated that the Cu-CDs have a substantial catalytic effect on ECR. And it is worthwhile to note the similarity of the Cu active sites on Cu-CDs with the Cu^2+^ chelated in CuPc for avoiding rapid reduction like Na_2_[Cu(EDTA)] at reducing potentials, which results from the heterogeneousness of Cu-CDs and CuPc in aqueous electrolyte.

The gaseous and liquid products of ECR on Cu-CD catalysts were analyzed by gas chromatography (GC) and ^1^H nuclear magnetic resonance (^1^H NMR) spectroscopy, respectively, and converted into Faradaic efficiencies (FEs) in Fig. 3b. It is worth noting that at all selected potentials, formic acid was the only detected liquid product with FE less than 1% (Supplementary Fig. 14). Therefore, we did not mark the FEs of formic acid in the diagram. In fact, trace amounts of CO and C_2_H_4_ as by-products can also be ignored. During a broadly applied potential, the Cu-CD electrode showed an unprecedented CO_2_ reduction selectivity to CH_4_, as more than 99% of the CO_2_ reduction products were CH_4_. Meanwhile, the FE(CH_4_) of Cu-CDs increased as a more negative potential was applied, from 42 ± 2% at −1.14 V to a maximum of 78 ± 2% at −1.44 V with total current density of ~40 mA/cm^2^, and subsequently, it declined to 55 ± 1% at −1.66 V (Fig. 3b). Similar single-atom Cu sites prepared by adding CuCl_2_ solution into pristine CDs (CDs+Cu^2+^, details in Methods) had the same CH_4_ selectivity as Cu-CDs whose by-products were prominently suppressed, except, a comparatively lower maximum FE(CH_4_) of 53.4 ± 0.8% at −1.44 V (Fig. 3c). It indicates that the CDs+Cu^2+^ shares the same active sites as Cu-CDs, and verified the coordinate interaction between copper ions and chelating ligands of CDs. We also confirmed carbon atoms in CH_4_ was from CO_2_ by using ^13^C isotopic-labeling method (Supplementary Fig. 28). In contrast, CuPc showed significantly lower FEs and poorer CH_4_ selectivity (Fig. 3d). The maximum FE(CH_4_) was only around 15% at −1.14 V with total current density of ~10 mA/cm^2^, and only 75% of the CO_2_ reduction products were CH_4_. As shown in Fig. 3e, the partial CH_4_ current density of Cu-CDs is 25 times higher than that of CuPc at an applied potential of −1.64 V where Cu-CDs reach the maximum turnover frequency (TOF) of 2370 h^-1^. These results indicated that the functional groups binding the Cu sites possess an ultra-stable +1 valence state, which prevents Cu species showing multi-valence so as to boost the selectivity of CH_4_^40^.

The calculation of stability of Cu-CDs was performed by continuous CO_2_ reduction at the highest FE(CH_4_) potential (−1.44 V, Fig. 3f). It is shown that after 6 h continuous test, the Cu-CDs could still remain 96% of the initial FE for CH_4_ formation, while the FE(CH_4_) of CuPc had apparent declines to only 4% after test. Afterward, the catalyst species on the Cu-CD electrode were washed with methanol and examined by HAADF-STEM. The image in Supplementary Fig 15 clearly shows that the Cu atoms remained atomically dispersed. Moreover, UV-visible and fluorescence spectroscopy (Supplementary Fig. 16a, b) were also applied to investigate the Cu-CDs after ECR process. Because of the photophysical properties that the CDs give intense fluorescent emission while Cu coordination quenches the fluorescence, the spectrum shows no fluorescence recovery of the Cu-CDs after electrocatalysis, illustrating the survival of single-atom Cu. The pattern of UV-visible spectrum also remains unchanged, which is in good accord with the fluorescence results. XPS study of post-electrolyzed Cu-CDs verified that the Cu^+^ state is stable in functional groups’ chelation after electrocatalysis (Supplementary Fig. 17).

### Origin of electrocatalytic activity

A set of control experiments were carried out to exclude the contribution from other species such as in situ formed Cu nanoparticles. Firstly, pristine CDs were tested for CO_2_ reduction under identical experimental conditions (Supplementary Fig. 18a, b). It was evident that the hydrogen dominated the products for the CD electrode at an applied potential of −1.14 to −1.64 V, which confirmed that the CDs only served as inert substrates to stabilize Cu ions. Besides, the result of CDs+Cu^2+^ (Fig. 3c) showed the same CH_4_ selectivity as Cu-CDs, suggesting the role of Cu ions.

Then, ex situ XRD and scanning electron microscopy (SEM) characterizations were carried out to reflect any structural change. As expected, there was no diffraction peak of Cu_2_O and Cu showed in the operated Cu-CD electrode (Supplementary Fig. 19c). SEM images showed a thin coat of Cu-CDs well dispersed on the surface of carbon fibers, and no existence of Cu_2_O or Cu was found after the stability test (Supplementary Fig. 19b). To exclude any neglected metallic particles formed between carbon paper fibers, we used a bare carbon paper as a cathode and directly added Cu-CDs into the electrolyte then quickly switched it while electrolyzed. A time course of FEs polarized at −1.44 V was recorded in Supplementary Fig 20. Due to Cu-CDs diffusion to the electrode, it turned out to have ~30% FE(CH_4_) for Cu-CDs dispersed in the electrolyte. Then we switched the electrode to fresh 0.5 M KHCO_3_ before 30 min, which showed a significant drop in FE. This experiment unambiguously confirmed that Cu or Cu_2_O was not deposited onto the cathode, and Cu-CDs were the catalyst that converts CO_2_ to CH_4_.

**Fig. 4:**
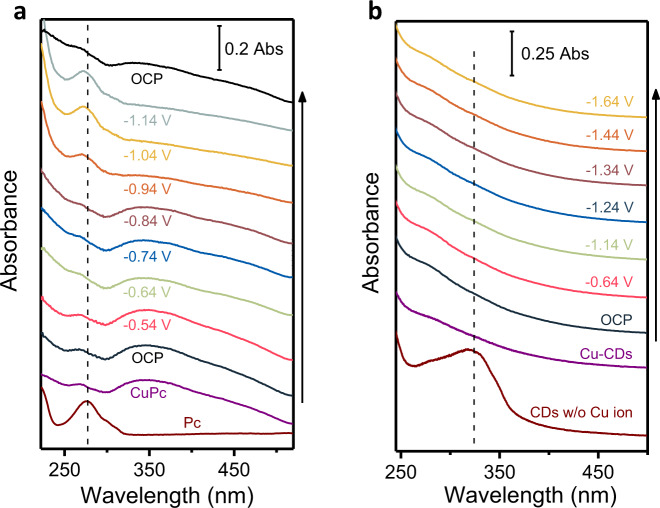
In situ UV-visible measurements under electrocatalytic reaction conditions. **a** In situ UV-visible spectra of CuPc. **b** In situ UV-visible spectra of Cu-CDs. The spectra were collected with transmission mode by depositing samples on the transparent electrode.

However, recent reports discovered that single-site Cu material could transiently convert into metallic Cu clusters that are considered as the active species for the catalysis under the working conditions. Intriguingly, this process is reversible upon release of the reduction potential^26,41^, which makes ex situ observation fail to detect Cu nanoparticles. To verify whether such restruction also occurred in our material, in situ UV-visible spectroscopy was applied under the ECR condition (details in Methods). Firstly, CuPc was deposited on a transparent platinum sputtered quartz plate whose potential was dropped in steps from open circuit potential, OCP = 0.86 V vs. RHE to –1.14 V vs. RHE. At each potential, a series of UV-light spectra were continuously collected until they did not exhibit any variation, so as to reflect electrochemical stable states. According to Fig. 4a, there was a palpable change of spectra pattern with lower potentials applied. Comparison of the operando spectra with those of reference CuPc and phthalocyanine (Pc) confirmed the decomposition of CuPc under ECR condition. Furthermore, it was worth noting that the spectra experienced two main changes: reducing CuPc absorption at −0.64 V and rising B-bands absorption of Pc at −0.94 V, corresponding to the formation of Cu(I) and Cu(0), respectively, which was reported by in situ XAS observations^26^. Accordingly, SEM characterizations were performed after the working electrode potential returned to 0.86 V (Supplementary Fig. 21). Like the previous study, well-dispersed nanoparticles emerged from cycled CuPc, which indicated that the same conversion took place during our operation. So we carried out in situ UV-visible measurements of Cu-CDs under the same conditions (Fig. 4b). Contrary to CuPc, decreasing electrode potential did not cause the shift of Cu-CD spectra to that of CDs without copper ion, and ex situ SEM did not observe any morphological changes (Supplementary Fig. 22). These experiments confirmed that it is single-site Cu that has intrinsic ECR activity in Cu-CDs rather than in situ formed Cu clusters.

### DFT calculations

**Fig. 5:**
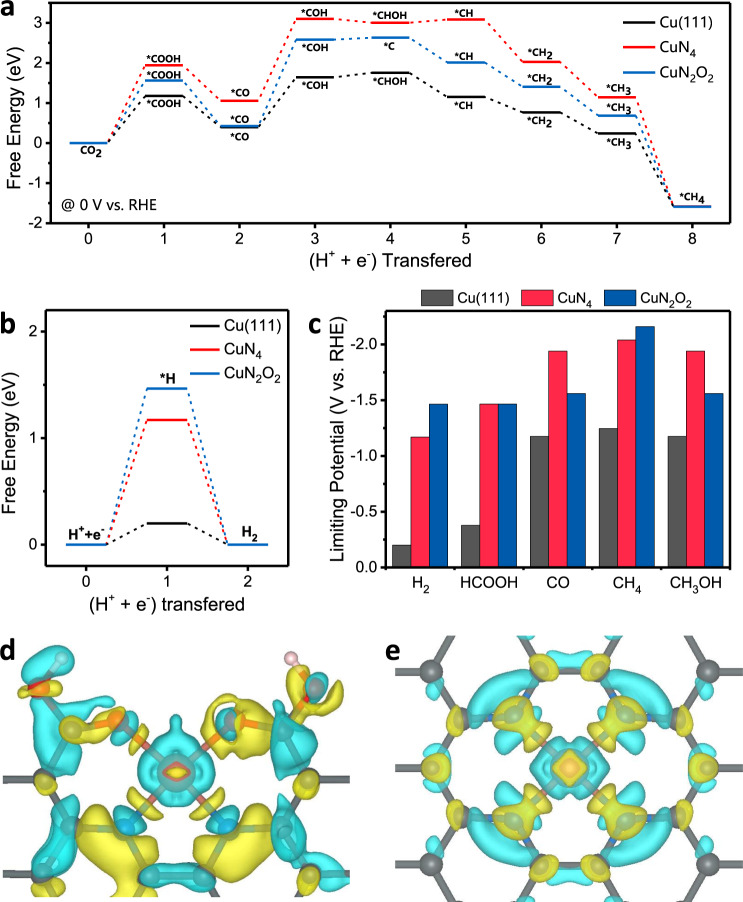
Evaluation of catalytic activity by DFT simulations. Free energy diagram of the: **a** CO_2_ reduction pathway to CH_4_ and **b** hydrogen evolution on the CuN_2_O_2_(Cu-CDs), CuN_4_, and Cu(111). **c** Summary of the limiting potentials of the products of ECR and HER on the CuN_2_O_2_, CuN_4_, and Cu(111). 3D differential charge densities of: **d** CuN_2_O_2_ and **e** CuN_4_. The yellow and blue isosurfaces correspond to the increase in the number of electrons and the depletion zone, respectively.

To further investigate the principles of structure–activity relationships laid in the CuN_2_O_2_ moieties of Cu-CDs, DFT calculations were performed. Firstly, we calculated the free energy of the lowest-energy pathways for electrochemical CO_2_-to-CH_4_ reduction on CuN_2_O_2_ in comparison with that of conventional CuN_4_ (Supplementary Fig. 23), and Cu(111) surface as a reference because the operando formed Cu clusters in CuPc. As shown in Fig. 5a, the introduction of CuN_2_O_2_ sites lowers the overall endothermic energy of intermediates for *COOH (1.56 eV) and *COH (2.58 eV) compared with that for CuN_4_ (1.94 eV and 3.10 eV, respectively). However, compared with the pathway on Cu(111) surface, the energy barriers are still higher for effectively processing the formation of CH_4_, which cannot explain the selectivity and activity advantages of Cu-CDs over CuPc.

However, further calculating the free energy profiles of H_2_ evolution reaction (HER) provided us a possible answer (Fig. 5b). The CuN_2_O_2_ sites show much higher formation energy for *H adsorption (1.46 eV) than that of CuN_4_ (1.17 eV) and Cu(111) (0.20 eV) surface, which indicates that HER is well inhibited by CuN_2_O_2_ moieties even under high negative bias. In addition, the free energy profiles for possible by-products were also investigated (Supplementary Fig. 24). Their limiting potentials (*U*_L_, *U*_L_ = −Δ*G*_max_/*ne*) were summarized in Fig. 5c. Generally, Cu(111) shows the lowest required potential to start multiple-electron reduction toward CO, CH_4_, and CH_3_OH. However, their limiting potentials are all around −1.2 V, which results in various ECR products once the reduction process starts. Also, the low *U*_L_ (−0.20 V) for H_2_ evolution is bound to cause much FE losses to competing HER on Cu(111) surface. All of these were reflected in the experimental results of CuPc. Conversely, CuN_2_O_2_ sites not only elevate the *U*_L_ of HER, but also differentiate the *U*_L_ of CH_4_ (−2.16 V) with that of HCOOH (−1.47 V), CO (−1.56 V), and CH_3_OH (−1.56 V). That makes the potential window of producing CH_4_ depart from other products’ to more negative bias so that CH_4_ can be exclusively produced at the potential with highest FE(CH_4_).

Furthermore, we also disclosed the reasons why CuN_2_O_2_’s copper center possesses low-valent oxidation state by calculating its differential charge densities which depicted a less positive-charge distribution density on the Cu atom of CuN_2_O_2_ than that of CuN_4_ (Fig. 5d, e). In addition, the calculated Bader charge uncovered that the CuN_2_O_2_ (+0.87*e*) carried less positive charge than CuN_4_ (+1.01*e*). Because CuN_4_ was previously proved to possess Cu(II) center^42^, we could infer CuN_2_O_2_ has Cu(I) oxidation state.

## Discussion

In summary, we have developed a new partial-carbonization approach to produce a well-controlled single atomic active sites on CDs. It consists unique CuN_2_O_2_ sites anchored at the edge of graphitic carbons for efficient and selective ECR. The as-synthesized Cu-CD catalyst enables electrochemical CO_2_ reduction to CH_4_ with high Faradaic efficiency of 78%, surpassing the current MN_*x*_C_4_ SACs. The superior catalytic CH_4_ selectivity at high negative bias originates from the new electronic structure of the CuN_2_O_2_ site, on which the HER is greatly suppressed, while the limiting step for CH_4_ production requires lower energy than other reported SACs. We believe this work not only promotes a novel and general strategy to design the metal coordination environment of SACs, but also provides an essential mechanism for rationally boosting specific product selectivity.

## Methods

### Synthesis of Cu-CDs and pristine CDs

Cu-CDs were synthesized according to a slightly modified version of a previously reported method^27^. In general, 1.6 g Na_2_[Cu(EDTA)]·2H_2_O was filled into a ceramic boat and placed in the center of a quartz tube, then calcined in a tube furnace at 250, 300, or 350 °C for 2 h at a heating rate of 5 °C/min under a N_2_ atmosphere. After pyrolysis, 80 mL anhydrous methanol was added to extract Cu-CDs from the product. The suspension was stirring for 15 min, then centrifuged twice at a high speed (11,000*g*) for 20 min to remove the insoluble copper and sodium salts. The upper brown solution was filtered with 0.22 μm Titan3™ PTFE syringe filters to remove carbon fragments. Pure Cu-CD powder was obtained by drying the methanol solution at 60 °C. Pristine CDs were synthesized from Na_2_[H_2_(EDTA)] precursor using the identical pyrolysis treatment at 250 °C.

### Electrochemical test

Cu-CD electrode and pristine CD electrode: To prepare Cu-CD electrode, 10 mg of Cu-CDs was dissolved in 1900 μL of methanol/water (1:1, v/v) mixed solvent and mixed with Nafion (100 μL, 5 wt%) followed by vortex for 5 min; 200 μL of catalyst ink was dropped onto the carbon paper (coated area: 7 mm × 7 mm × 2, loading: 1.0 mg/cm^2^) and dried overnight at 60 °C in a vacuum oven. CD electrode was prepared under the same procedure.

CuPc electrode: 40 mg of CuPc was mixed with 200 mL of trichloromethane by sonication for more than 30 min to form homogeneous inks. For each electrode, 200 µL of the ink was dropped onto the carbon paper (coated area: 7 mm × 7 mm × 2, loading: 40 μg/cm^2^) and dried overnight at 60 °C in a vacuum oven.

CDs+Cu^2+^ electrode: 10 mg of pristine CDs was dissolved in 1900 μL of methanol/water (1:1, v/v) mixed solvent then mixed with Nafion (100 μL, 5 wt%) and 10 μL of CuCl_2_ solution which was prepared by adding 9.3 mg of CuCl_2_ in 1 mL methanol, followed by vortex for 5 min; 200 μL of catalyst ink was dropped onto the carbon paper (coated area: 7 mm × 7 mm × 2, loading: 1.0 mg/cm^2^) and dried overnight at 60 °C in a vacuum oven.

Na_2_[Cu(EDTA)] electrode: 190 μL of the Na_2_[Cu(EDTA)] solution, which was prepared by adding 30 mg Na_2_[Cu(EDTA)] in 190 mL methanol/water (1:1, v/v) mixed solvent, was mixed with Nafion (10 μL, 5 wt%) and dropped onto the carbon paper (coated area: 7 mm × 7 mm × 2, loading: 30 μg/cm^2^) and dried overnight at 60 °C in a vacuum oven.

CO_2_ reduction experiments were performed in aqueous 0.5 M KHCO_3_ solution saturated with CO_2_/N_2_ in a twin-cell with nafion-117 membrane. All potentials were converted to those against a RHE reference:

*E* (vs. RHE) = *E* (vs. Ag/AgCl) +0.197 V + 0.0592 pH V

Each compartment of the twin cell contained 8 mL electrolyte and approximately 10 mL headspace. The electrolytes were purged with CO_2_ for 30 min (pH = 7.8) under stirring to ensure saturation before tests. With an Alicat gas controller, the flow rate of CO_2_ was controlled at 15 mL/min (standard cm^3^/min) and routed directly into the gas sampling loop of GC. The gas phase composition was analyzed by GC every 10 min and liquid products were analyzed by ^1^H NMR after test of each applied potential.

### Evaluation of TOF

The TOF for CO was calculated as follows:

1 $${\mathrm{TOF}} = \frac{{I_{{\mathrm{product}}}/NF}}{{m_{{\mathrm{cat}}} \times M_{{\mathrm{Cu}}}}} \times 3600$$

*I*_product_: partial current for certain product, CH_4_;

N: the number of electron transferred for product formation, which is 8 for CH_4_;

F: Faradaic constant, 96,485 C/mol;

*m*_cat_: loaded catalyst mass in the electrode, g;

*ω*: Cu loading in the catalyst, wt%;

M_Cu_: atomic mass of Cu, 63.55 g/mol.

### In situ UV-visible spectroscopy

To carry out in situ UV-visible spectroscopy, a 10 mm quartz cell was employed as an electrochemical cell. The cell was filled with electrolyte (0.5 M aqueous KHCO_3_). Ag/AgCl and Pt gauze were used as reference electrode and counter-electrode, respectively. The working electrode is a 9 mm × 50 mm quartz plate with a thin Pt layer by magnetron sputtering. The catalyst ink was dropped onto the quartz plate and dried overnight at 60 °C in a vacuum oven. The schematic illustration is shown in Supplementary Fig 26. During the measurement, a series of potentials were applied to the working electrode.

## Supplementary information

Supplementary Information

## Data Availability

The data that support the findings of this study are available within the paper and its Supplementary Information file or are available from the corresponding authors upon request. Source data are provided with this paper.

## Source data

Source Data
