# Supplementary material for: Insights on forming N,O-coordinated Cu single-atom catalysts for electrochemical reduction CO2 to methane
Source: Nat Commun. 2021 Jan 26;12:586. doi: 10.1038/s41467-020-20769-x (PMC7838205; doi:10.1038/s41467-020-20769-x)
Supplement: Supplementary file 1 — Supplementary Information [file 41467_2020_20769_MOESM1_ESM.pdf]

Insights on forming N,O-Coordinated Cu single-atom catalysts for electrochemical reduction CO_2 to Methane

Yanming Cai[1,3], Jiaju Fu[1,3], Yang Zhou[2,3], Yu-Chung Chang[2], Qianhao Min[1], Jun-Jie Zhu[1], Yuehe Lin[2*], Wenlei Zhu[2*]*

[1] State Key Laboratory of Analytical Chemistry for Life Science, School of Chemistry and Chemical Engineering, Nanjing University, Nanjing 210023, P. R. China

[2] School of Mechanical and Materials Engineering, Washington State University, Pullman, WA 99164, USA

[3] These authors contributed equally: Y. Cai, J. Fu and Y. Zhou.

Supplementary Methods.

Materials. Ethylenediaminetetraacetic acid copper disodium salt ($Na_2[Cu(EDTA)]$) and ethylenediaminetetraacetic acid disodium salt ($Na_2[H_2(EDTA)]$) were purchased from Shanghai yuanye Bio-Technology Co., Ltd. potassium bicarbonate (≥99.99% trace metals basis, 99.7-100.5% dry basis) and methanol (≥99.9%) were from Aladdin. Nafion perfluorinated resin solution (5 wt%) and Copper(II) chloride (99%) were purchased from Sigma Aldrich. CuPc (α-type, ≥90%) were purchased from TCI. Toray Carbon Paper (TGP-H-60) was from Fuel Cell Store. All reagents were of analytical grade and used without further purification. Nitrogen (99.99%) and carbon dioxide (99.99%) were purchased from Tianze gas, Inc. Deionized water from a Millipore Auto pure system was used as solvent.

Characterizations. The high-resolution transmission electron microscopy (HR-TEM) were observed on a JEOL JEM-2800 (200 kV). The high-angle annular dark-field scanning transmission electron microscopy (HAADF-STEM) characterization was performed on a FEI Titan^3 G2 STEM with a spherical aberration corrector. The X-ray absorption spectra were collected on the beamline BL01C1 in NSRRC, which using a Si (111) double crystal monochromator to record data. XANES and EXAFS data reduction and analysis were processed by Athena software. SEM measurements were performed with a JEOL JSM-7800F. FT-IR spectra were recorded on a Thermo Scientific NICOLET iS10. XPS was determined by a Thermo Scientific K-Alpha+ X-ray photoelectron spectrometer using Al Kα radiation. UV-Vis spectra were recorded on a Shimadzu UV-3600 spectrophotometer. Electrochemical measurements were performed with the Ivium CompactStat.h20250 electrochemical workstation using a three-electrode system with Ag/AgCl (filled with 3M KCl) as a reference and Pt plate as a counter electrode. Agilent 7890B gas chromatography (GC) was used to analyzing the gaseous products. ^{1}HNMR spectra were recorded on Bruker DRX 400 Avance MHz spectrometer. Solution pH was measured with the Thermo Scientific Orion VERSA STAR pH Benchtop Meter.

Isotope experiment methods. The ^{13}C-labeled $^{13}CO_2$ was purchased from Tianze gas.

The same electrolysis conditions was conducted except using $^{13}CO_2$ to purge $KHCO_3$ solution. The applied potential was set at -1.44 V and the gas products were transferred to upside-down 10 mL cramp-type headspace vial through water gas displacing. Gas chromatography-mass spectrometry (GC-MS, Agilent, 7890B-7000C MS) with a quadrupole-type mass spectrometer and HP-PLOT Q column (Agilent) was used for the measurements of the mass of the products. 500 μL of the gas was injected into the GC-MS by a syringe. The mass of products was analyzed after segregation by a gas chromatograph.

Computational methods. All the calculations was performed within the framework of the density functional theory (DFT) as implemented in the Vienna Ab initio Software Package (VASP 5.3.5) code within the Perdew–Burke–Ernzerhof (PBE) generalized gradient approximation and the projected augmented wave (PAW) method [1-4] . The cutoff energy for the plane-wave basis set was set to 400 eV. The Brillouin zone of the surface unit cell was sampled by Monkhorst–Pack (MP) grids for catalyst structure optimizations [5] . A 4 × 3 supercell of the graphene surface including 4 atomic layers was constructed to model the catalyst in this work. The graphene surface was determined by 3 × 3 × 1 Monkhorst−Pack grid. The convergence criterion for the electronic self-consistent iteration and force was set to 10^{-5} eV and 0.01 eV/Å, respectively. A vacuum layer of 12 Å was introduced to avoid interactions between periodic images.

The free energies of adsorbates and transition states at temperature T were estimated according to the harmonic approximation, and the entropy is evaluated using the following equation:

$$S(T) = k_B \sum_{i}^{\text{harm DOF}} \left[\frac{\varepsilon_i}{k_B T(e^{\varepsilon_i/k_B T} - 1)} - \ln(1 - e^{-\varepsilon_i/k_B T}) \right] \quad (1)$$

where k_B is Boltzmann's constant and DOF is the number of harmonic energies (ε_i) used in the summation denoted as the degree of freedom, which is generally 3N, where N is the number of atoms in the adsorbates or transition states. Meanwhile, the free energies of gas phase species are corrected as:

$$G_g(T) = E_{elec} + E_{ZPE} + \int C_p \, dT - TS(T) \quad (2)$$

where C_p is the gas phase heat capacity as a function of temperature derived from

Shomate equations and the corresponding parameters in the equations were obtained from NIST.

The limiting potential (U_L) is important factors for evaluating the catalytic activity, which represents the theoretically smallest negative potential at which the pathway becomes exergonic. The limiting potential is obtained using the formula:

$$U_L = -\Delta G_{max}/ne \tag{3}$$

where ΔG_{max} is the relative change of the Gibbs free energy of the rate-determining step.

Supplementary Discussion.

Faradaic efficiency. During electrochemical CO_2 reduction measurements, we found that some of the total FE values are higher than 100%. It is attributed to measuring error, such as current error, gas flow rate error, and gas chromatography quantitative error. The gas flow rate error is the most influential one, because the CO_2 feedstock will partially dissolve in the electrolyte so the gas flow rate at the electrolytic cell outlet will fluctuate. The change of gas flow rate will change the dilution ratio of products, which introduces error when we calculate the amount of products. To prove this, a control experiment was performed using the N_2 to replace CO_2 so as to eliminate the feedstock dissolution. Thus, the gas flow rate at the electrolytic cell outlet will not fluctuate, and the only production is H_2. As we can see from the result (Supplementary Fig. 29), all the FE values are below 100% but quite close to 100%. Theoretically, the total FE should be exactly 100%, because every electron needs faradaic process to cross electrolyte in steady state. Therefore, the FEs should fluctuate around 100% when there is measuring error in the system.

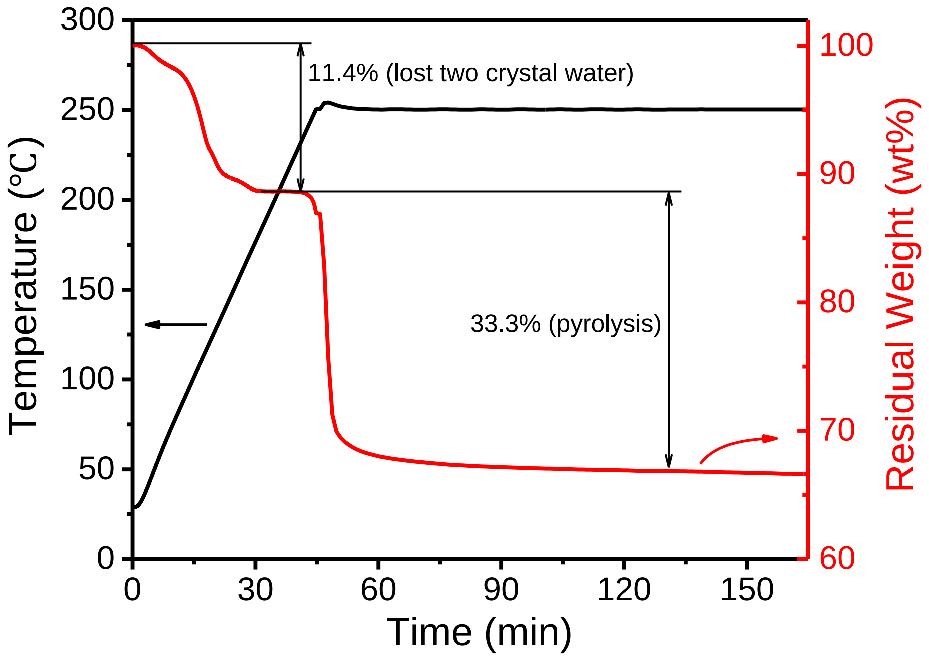

Supplementary Fig. 1. TGA curves of $Na_2[Cu(EDTA)]\cdot 2H_2O$. The TGA curve exhibits the crystal water weight loss of 11.4% below 250 °C, which is in line with the weight percentage of two water molecules in $Na_2[Cu(EDTA)]\cdot 2H_2O$. The second platform of 33.3% weight loss after reaching 250 °C represents the pyrolysis of EDTA ligand.

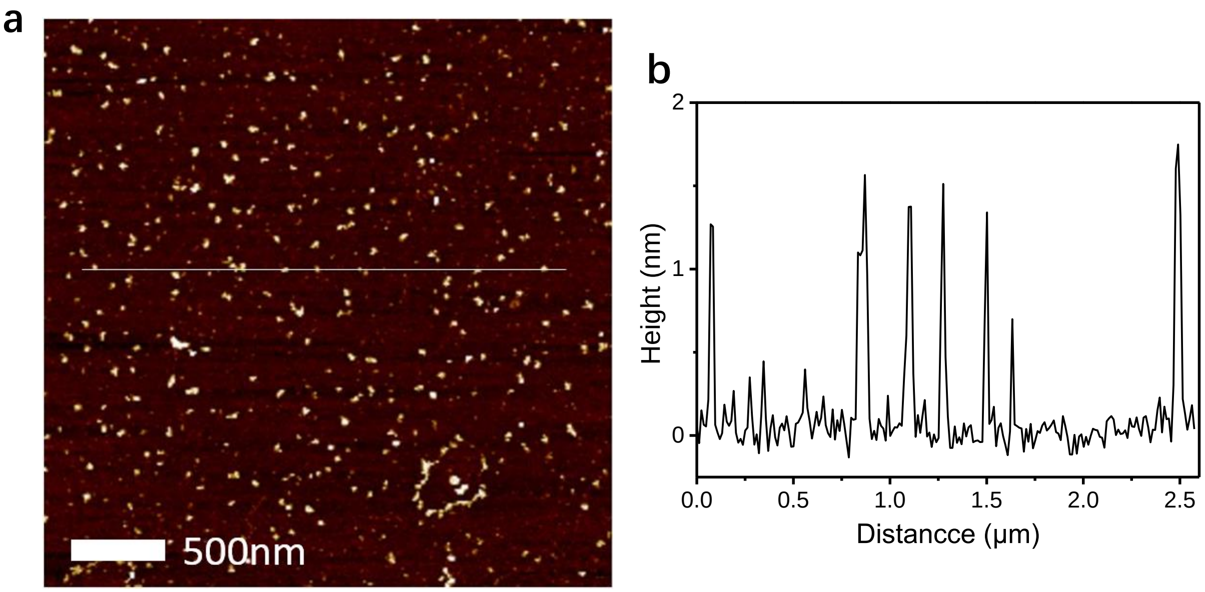

Supplementary Fig. 2. Morphology of Cu-CDs. (**a**) AFM image of Cu-CDs. (**b**) Thickness profiles. The obtained Cu-CDs had a thickness between 0.7 and 1.8 nm, corresponding to one to three atomic layers.

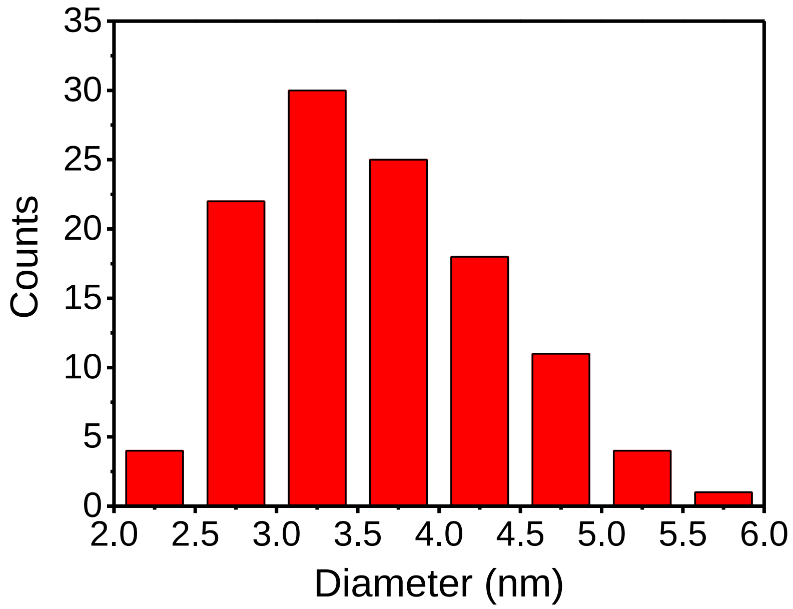

Supplementary Fig. 3. Statistical diameter of Cu-CDs.

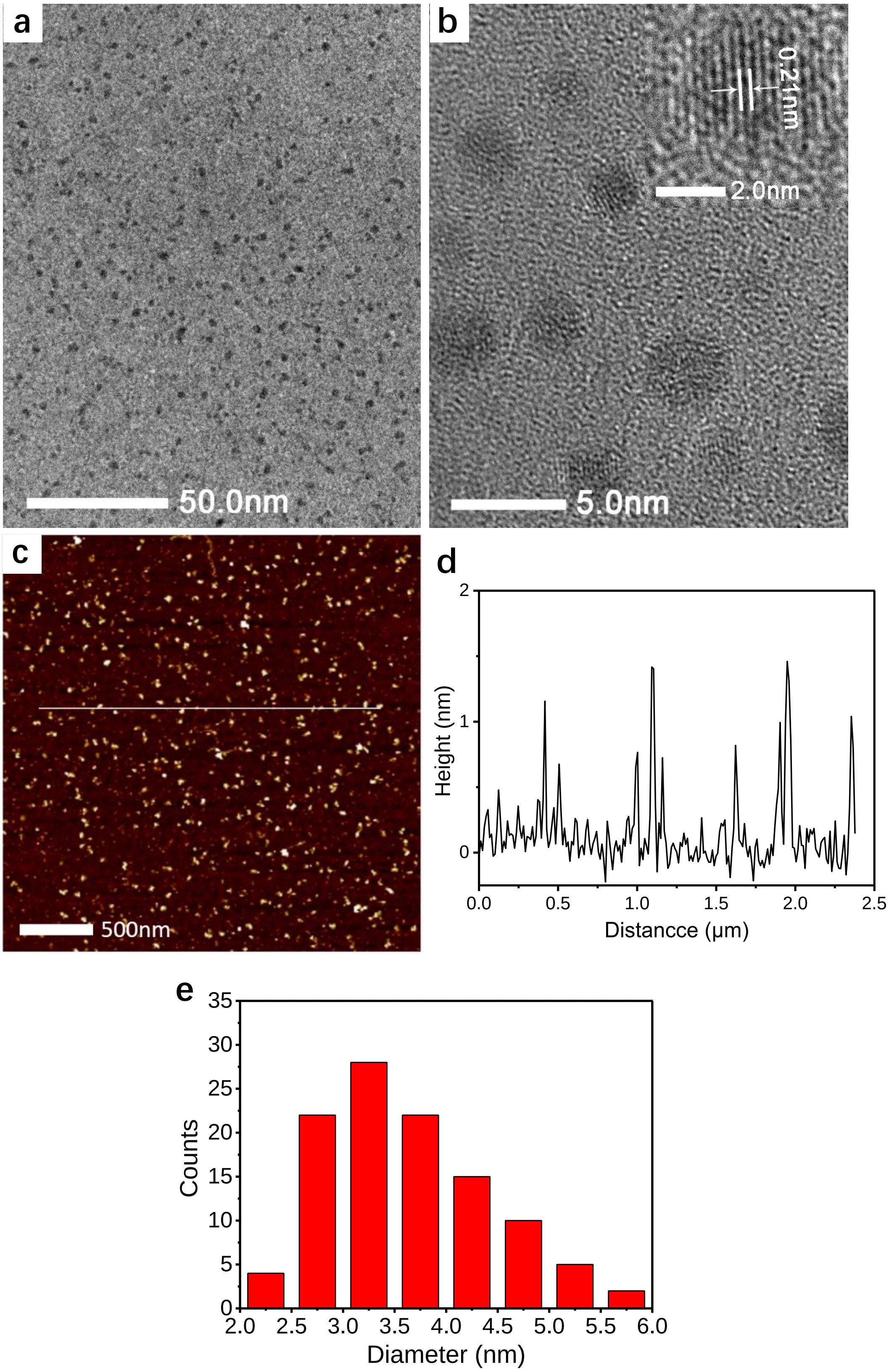

Supplementary Fig. 4. Morphology of CDs. (**a-b**) HRTEM image of pristine CDs. (**c**) AFM image of pristine CDs. (**d**) Thickness profiles. (**e**) Statistical diameter.

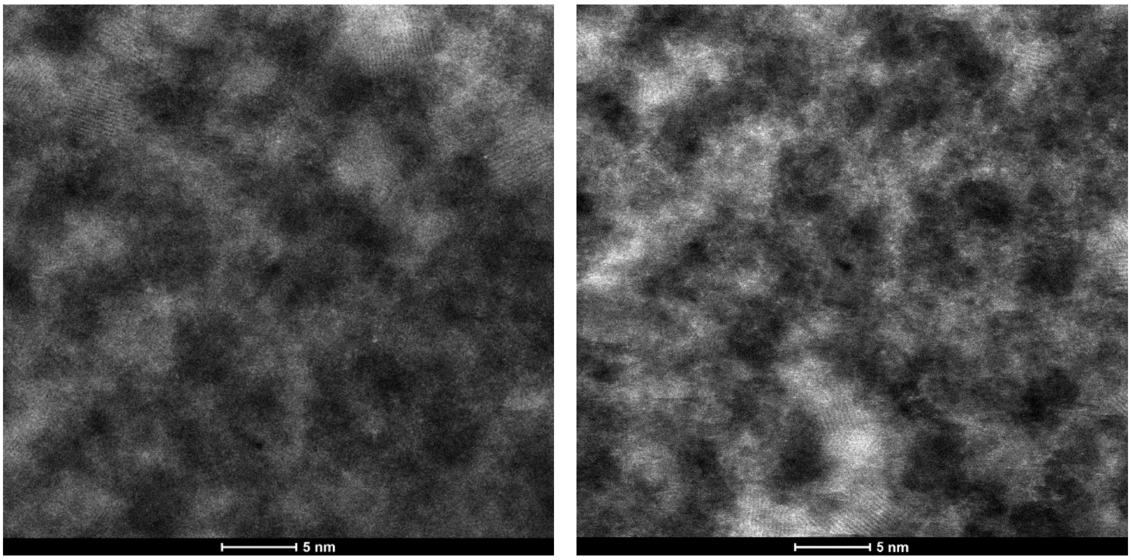

Supplementary Fig. 5. HAADF-STEM images of Cu-CDs.

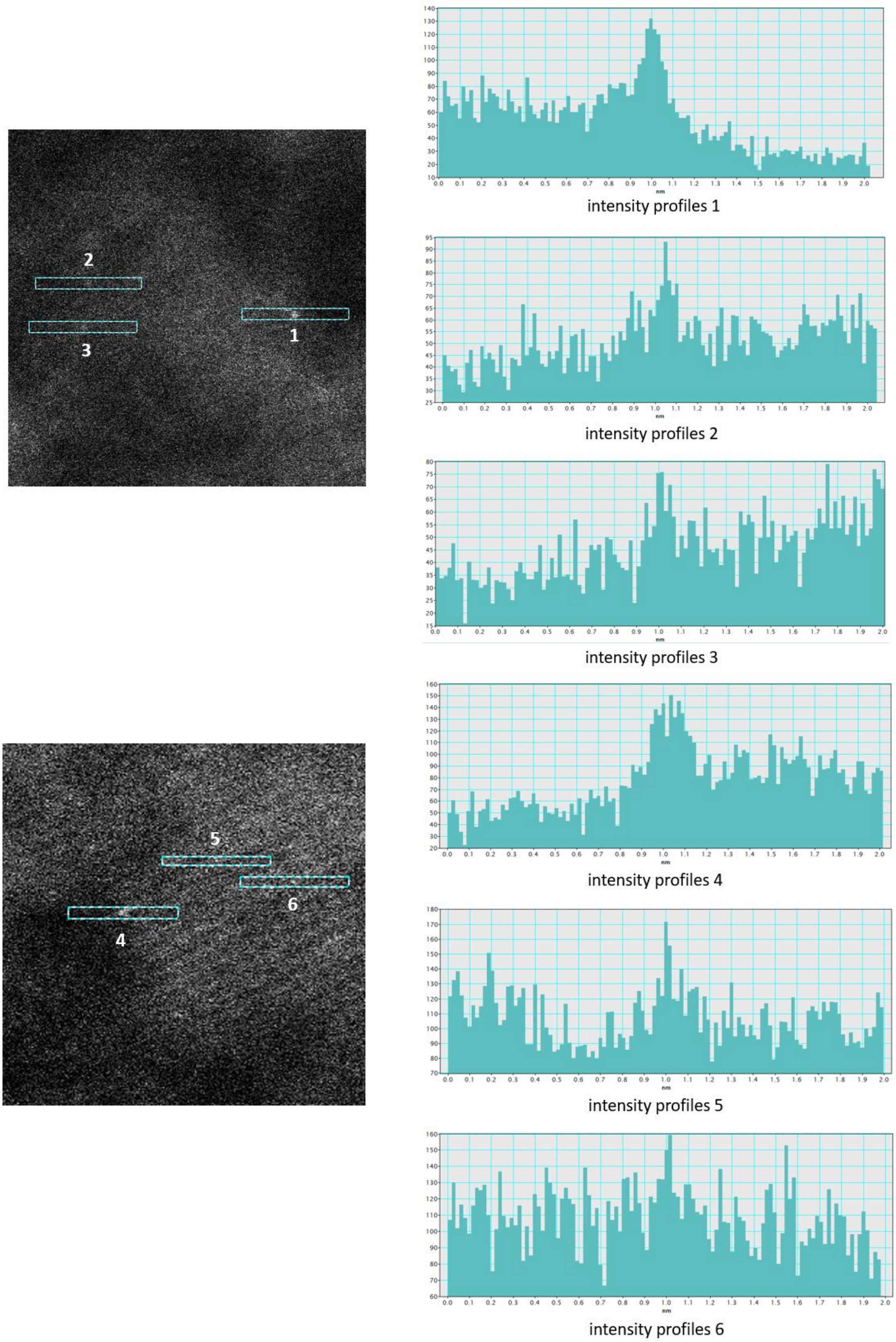

Supplementary Fig. 6. The corresponding intensity profiles of HAADF-STEM bright spots labeled 1-6. The diameter of bright spots 1-4 are in the range of isolated Cu atom size level (~0.28 nm reported in https://periodic.lanl.gov/29.shtml). The diameter of bright spots 5,6 are too small to be identified as single atoms (0.06 – 0.6 nm reported in https://en.wikipedia.org/wiki/Atomic_radius).

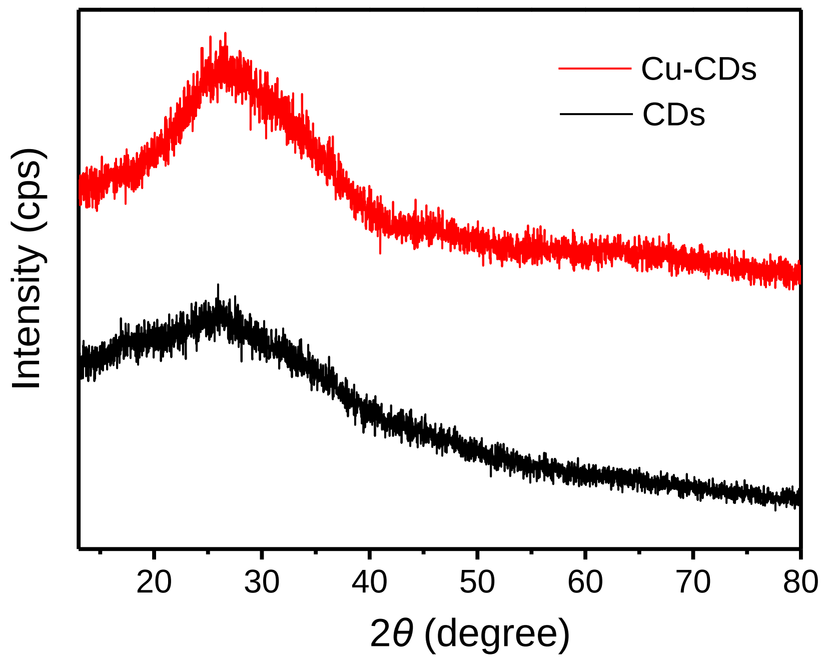

Supplementary Fig. 7. XRD patterns of fresh Cu-CDs and CDs.

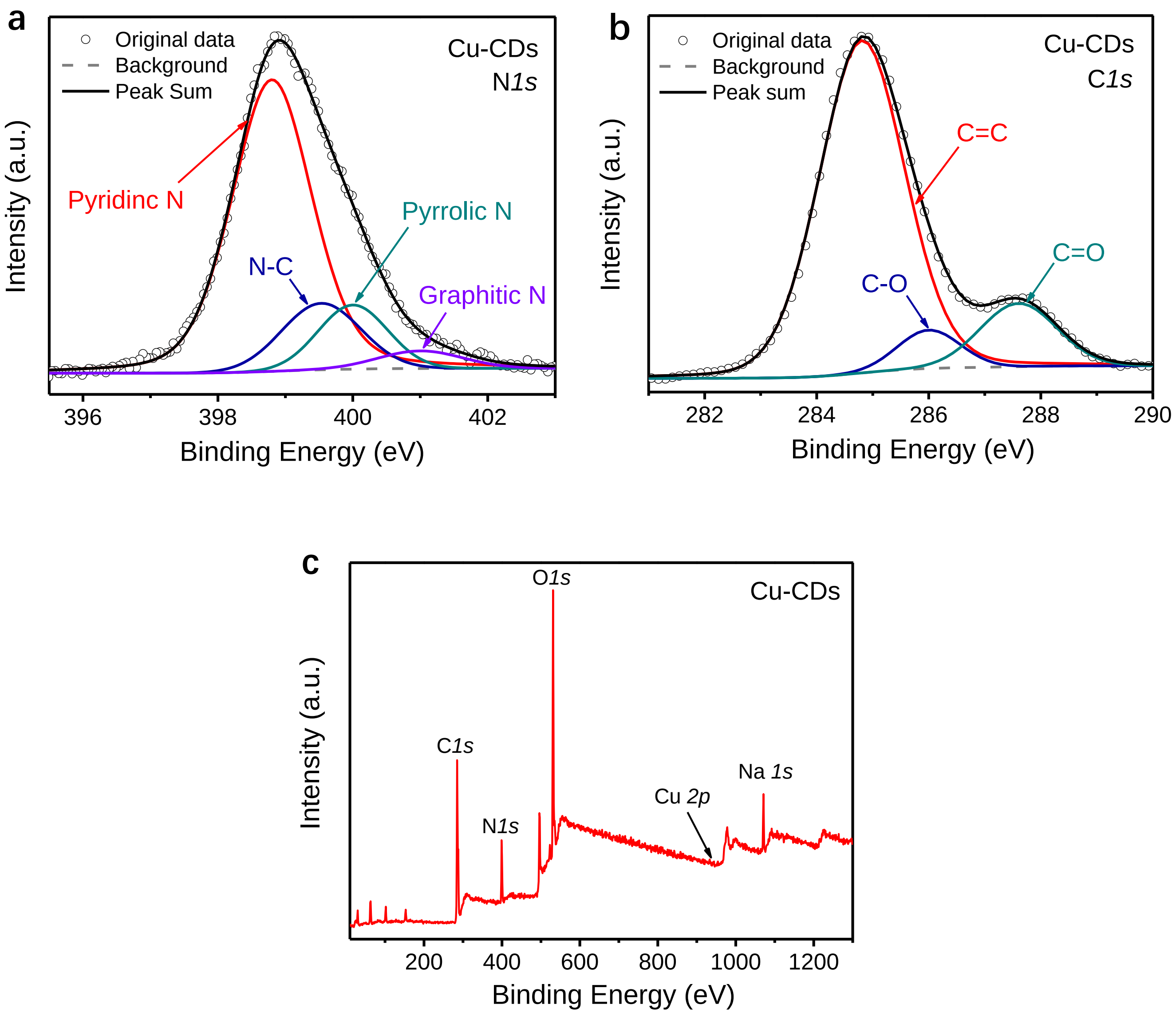

Supplementary Fig. 8. XPS spectra of Cu-CDs. **(a)** High-resolution N *1s* spectrum for Cu-CDs, deconvoluted into four sub-peaks representing pyridinic N (398.8 eV), N-C (399.5 eV), pyrrolic N (400.0 eV) and graphitic N (401.0 eV), **(b)** High-resolution C *1s*, **(c)** XPS survey spectra.

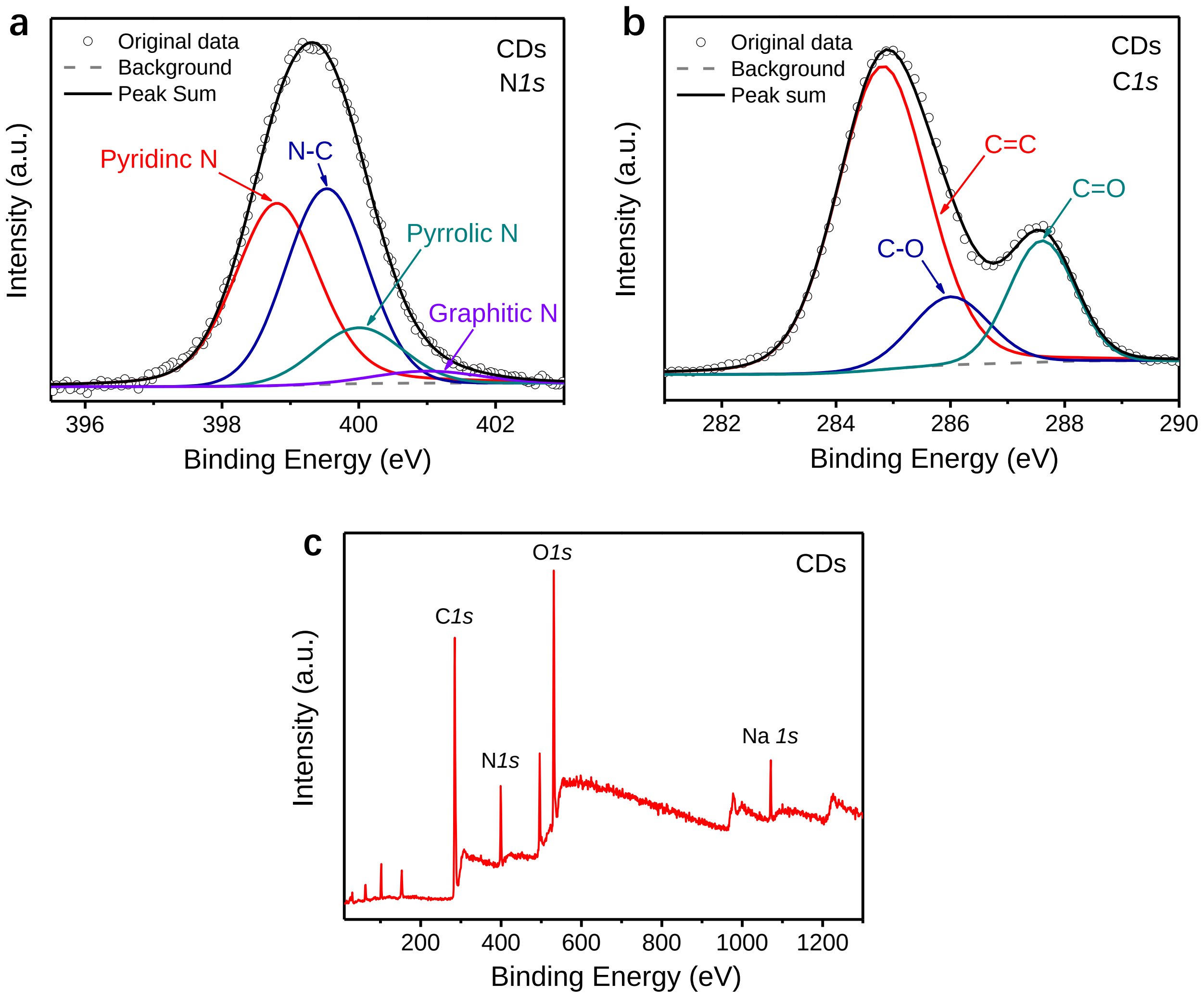

Supplementary Fig. 9. XPS spectra of CDs. **(a)** High-resolution N *1s* spectrum for CDs, deconvoluted into four sub-peaks representing pyridinic N (398.8 eV), N-C (399.5 eV), pyrrolic N (400.0 eV) and graphitic N (401.0 eV), **(b)** High-resolution C *1s*, **(c)** XPS survey spectra.

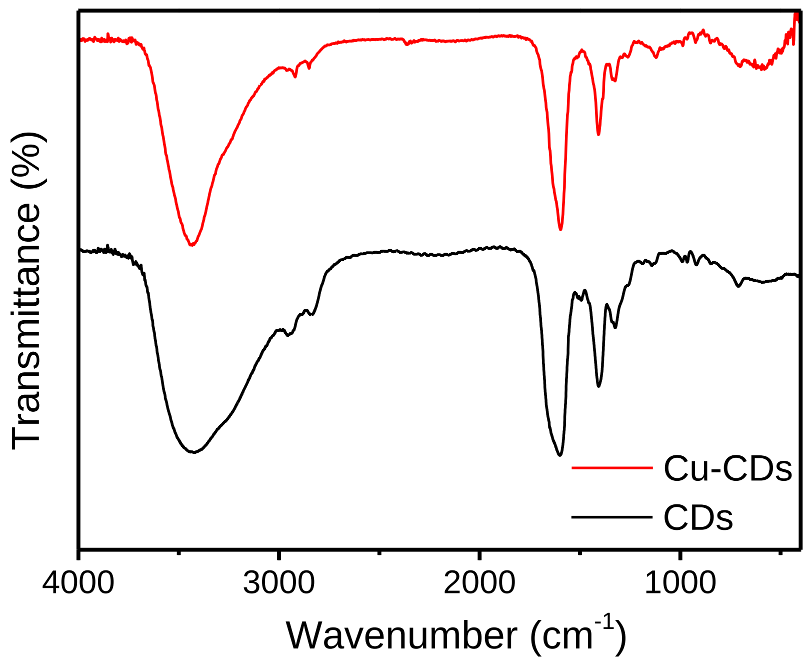

Supplementary Fig. 10. Comparison of FT-IR spectra of CDs and Cu-CDs.

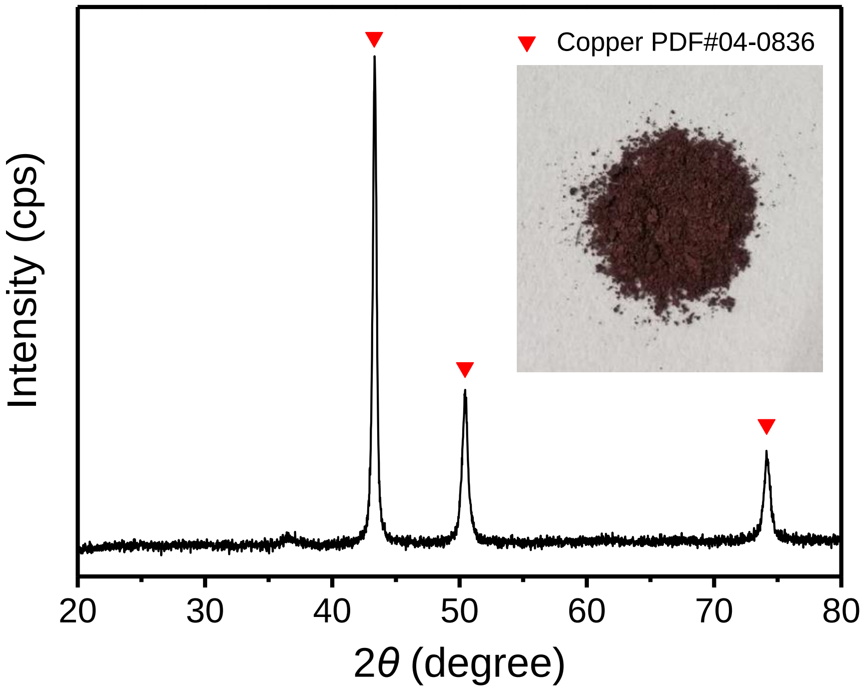

Supplementary Fig. 11. XRD pattern of the undissolved material after $Na_2[Cu(EDTA)]$ pyrolysis. The inset shows the photograph of undissolved solid. The exist of Cu^0 means that Cu^{2+} ions in $Na_2[Cu(EDTA)]$ have oxidizing ability. Thus, the EDTA's N with -3 valence state tends to be oxidized to pyridine N with -2 valence state in Cu-CDs, while the pyrrolic N and N-C with -3 valence state are dominated in pristine CDs.

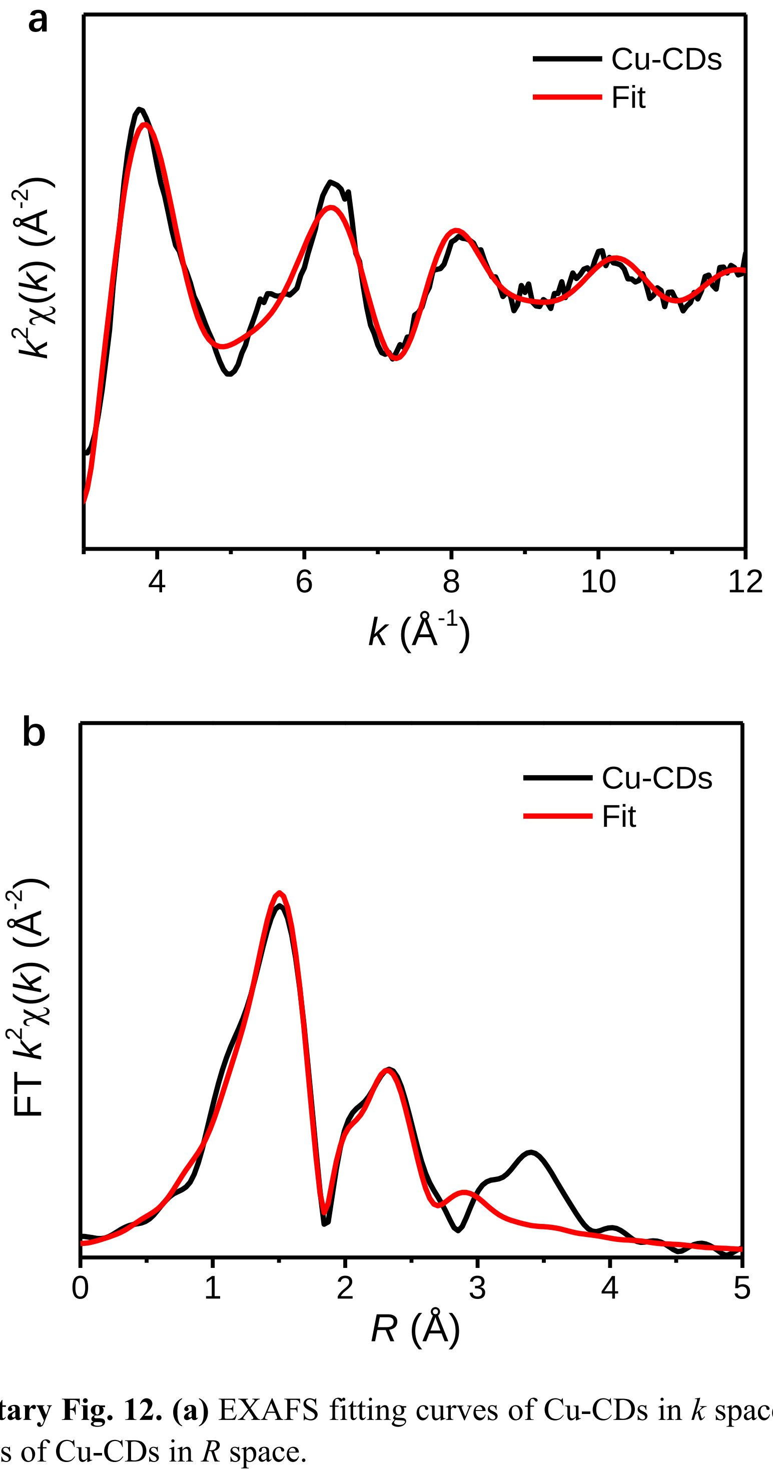

Supplementary Fig. 12. (a) EXAFS fitting curves of Cu-CDs in *k* space, **(b)** EXAFS fitting curves of Cu-CDs in *R* space.

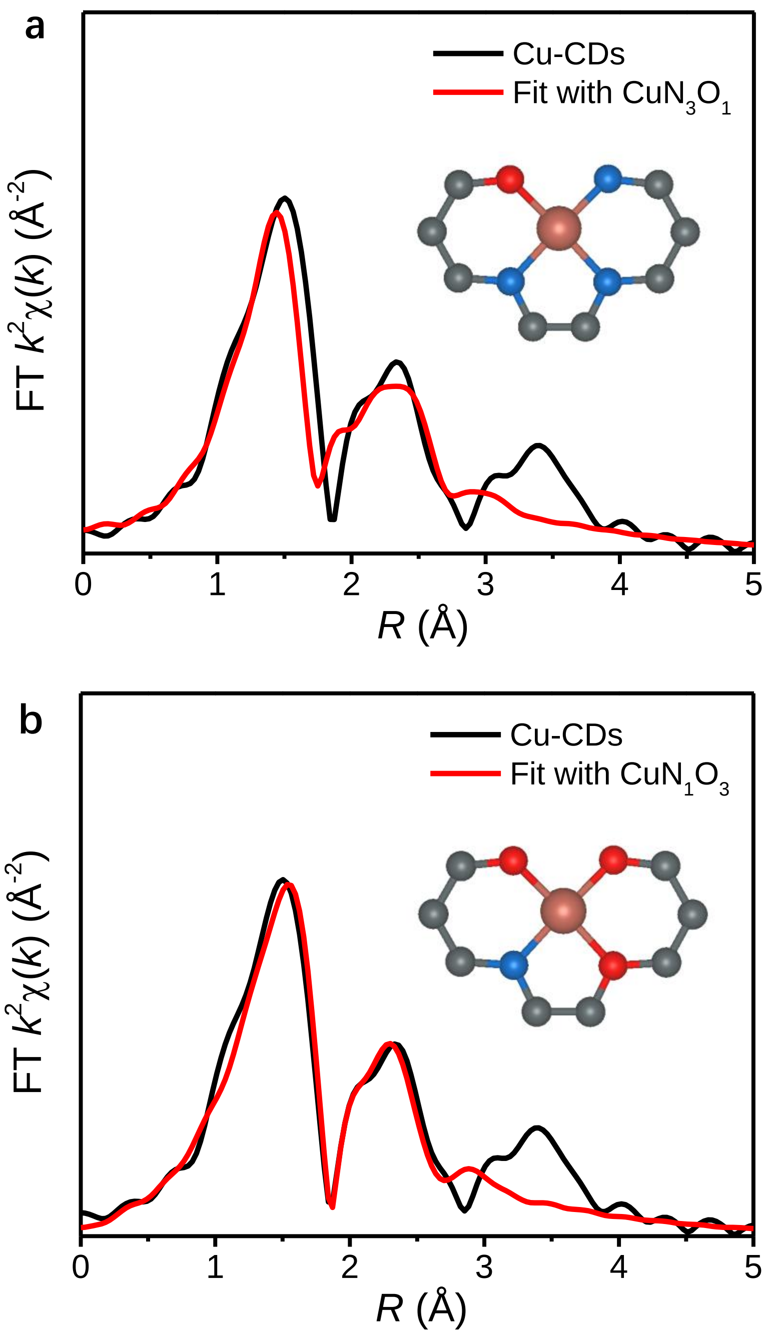

Supplementary Fig. 13. (a) EXAFS fitting curves of CuN_3O_1 models in *R* space, **(b)** EXAFS fitting curves of CuN_1O_3 models in *R* space.

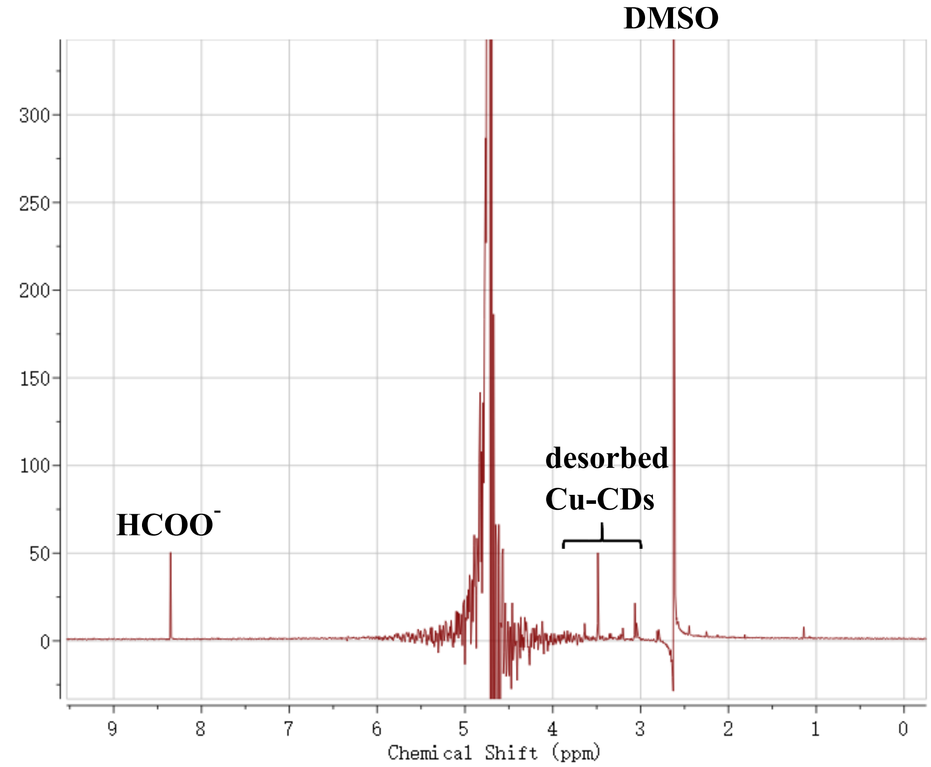

Supplementary Fig. 14. Example of NMR spectra of the electrolyte after CO_2 reduction electrolysis for Cu-CDs. DMSO is used as an internal standard for quantification of formate.

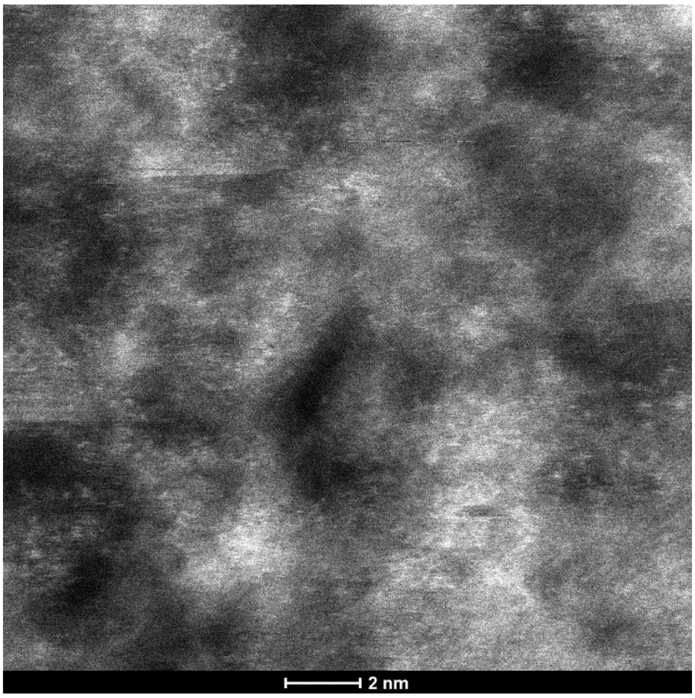

Supplementary Fig. 15. HAADF-STEM image for the single-atomic Cu on Cu-CDs after ~6 h CO_2 electroreduction.

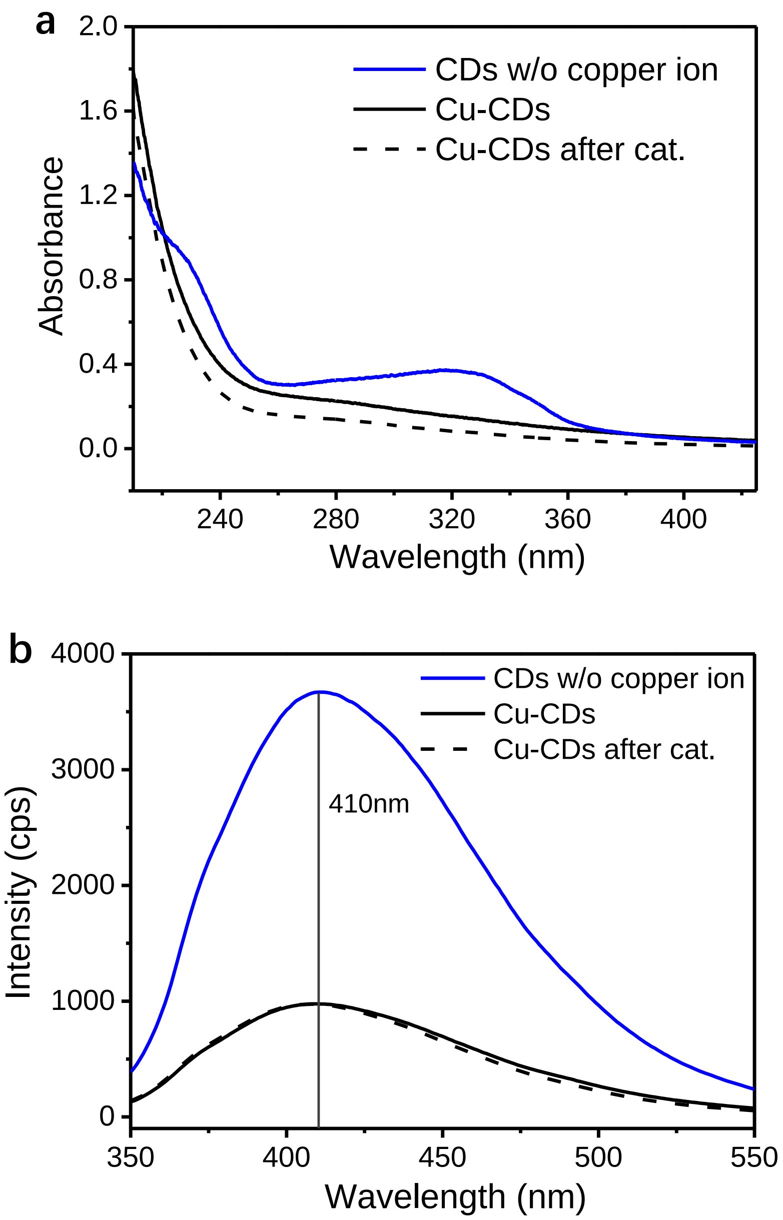

Supplementary Fig. 16. (a) UV-Vis absorption for Cu-CDs without Cu^{2+} ion, Cu-CDs and Cu-CDs after electrolysis at –1.44 V vs. RHE for ~6 h. The spectra were collected under transmission mode in a cuvette and we kept those samples at the same concentration. **(b)** fluorescence spectra excited by 330 nm excitation for Cu-CDs without Cu^{2+} ion, Cu-CDs and Cu-CDs after electrolysis at –1.44 V vs. RHE for ~6 h.

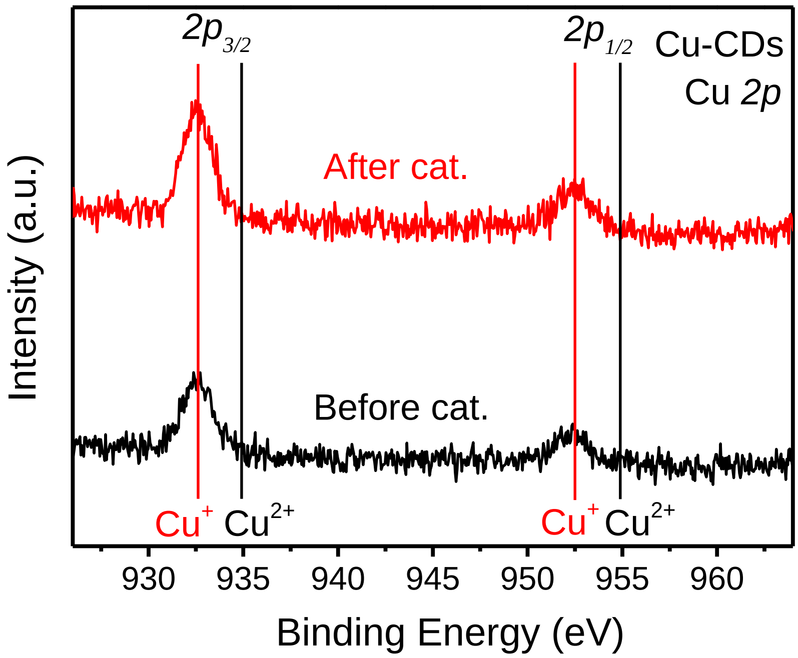

Supplementary Fig. 17. High-resolution Cu *2p* spectrum for Cu-CDs before and after electrolysis at –1.44 V vs. RHE for ~6 h.

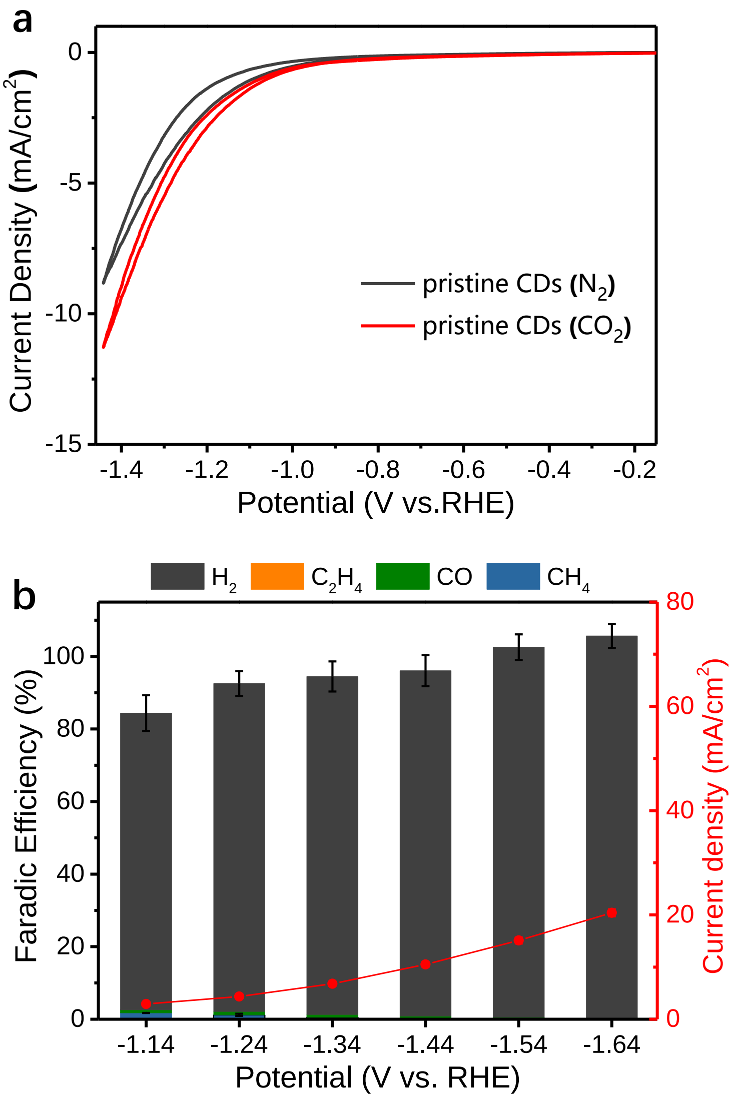

Supplementary Fig. 18. (a) CV curves of pristine CDs recorded in N_2 and CO_2–saturated 0.5 M $KHCO_3$ electrolyte with 10 mV/s scan speed. **(b)** Dependence of FE (left Y-axis) and current density (based on geometric surface area, right Y-axis) on the applied potential for pristine CDs.

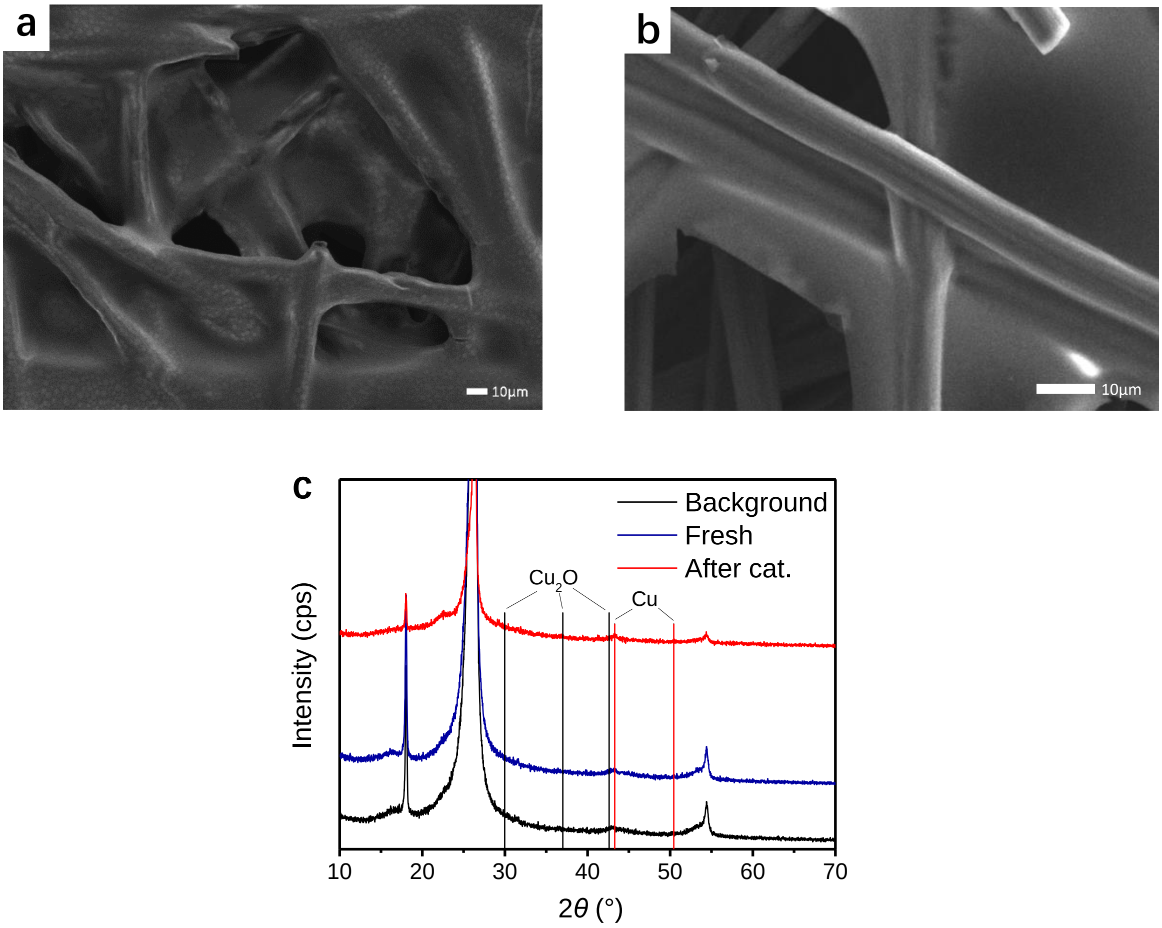

Supplementary Fig. 19. SEM and XRD characterizations of the Cu-CDs catalyst electrode before and after electrolysis at –1.44 V vs. RHE for ~6 h. (**a**) fresh electrode, (**b**) electrode after electrolysis, (**c**) XRD patterns.

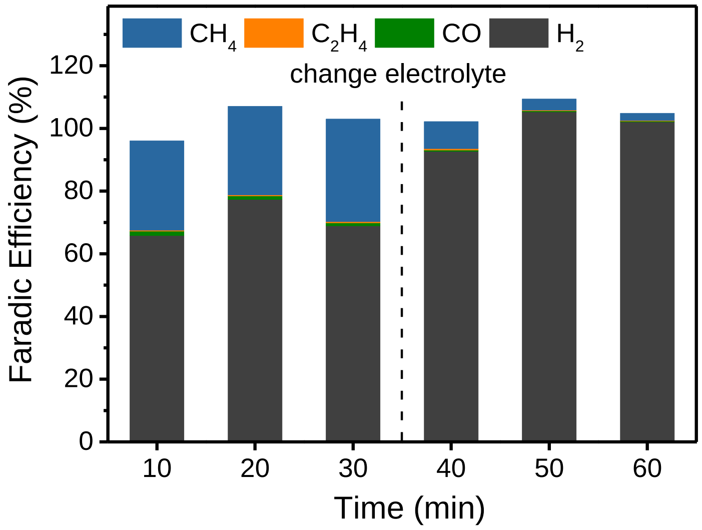

Supplementary Fig. 20. Control experiment of Cu-CDs dispersed in electrolyte. 200 μL of Cu-CDs catalyst ink was dispersed in 8 mL catholyte and bare CP was employed as electrode and polarized at −1.44 V for 30 mins, then quickly switched the electrode to fresh CO_2-saturated 0.5 M $KHCO_3$ without Cu-CDs at 30 min. Another 30 min electrolysis was tested after switch.

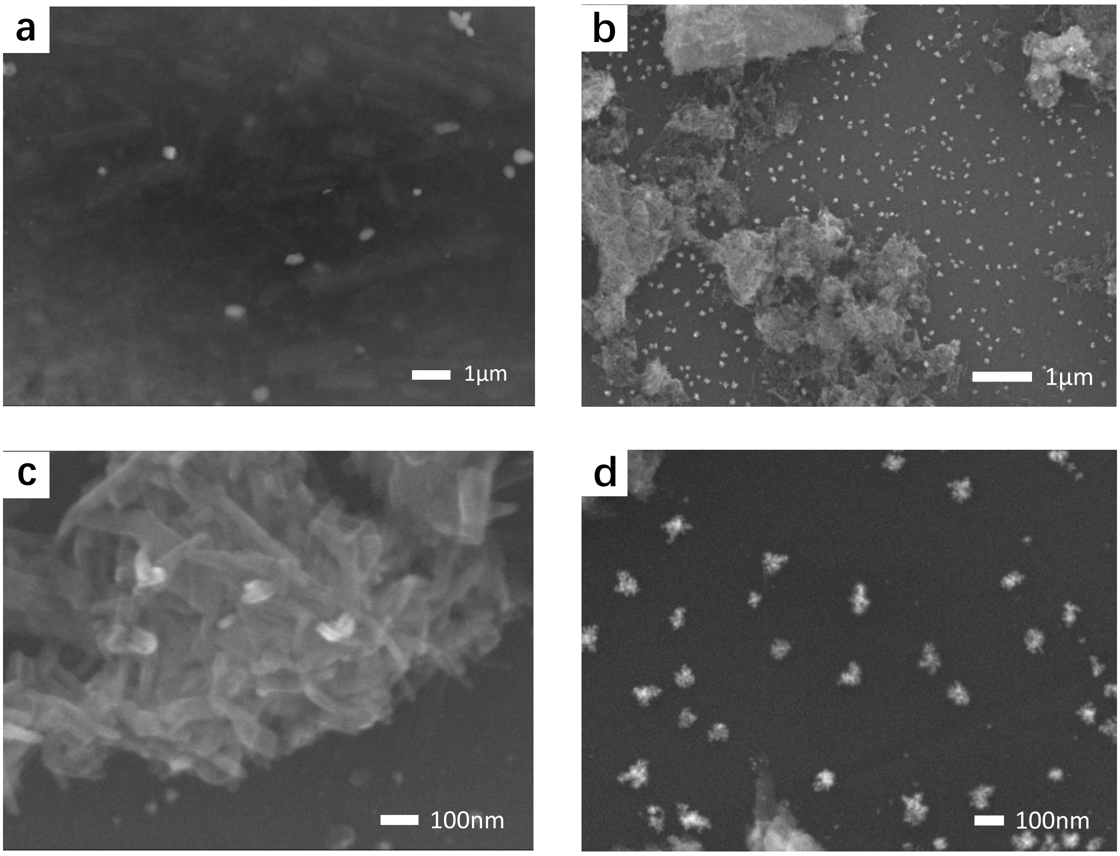

Supplementary Fig. 21. SEM characterizations of CuPc on Platinum sputtered quartz plate (**a**) before and (**b-d**) after electrolysis.

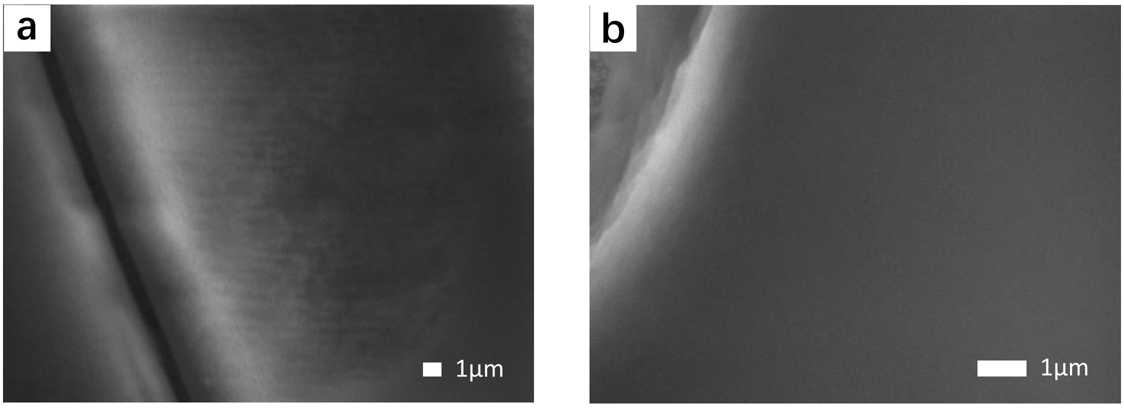

Supplementary Fig. 22. SEM characterizations of Cu-CDs on Platinum sputtered quartz plate (**a**) before and (**b**) after electrolysis.

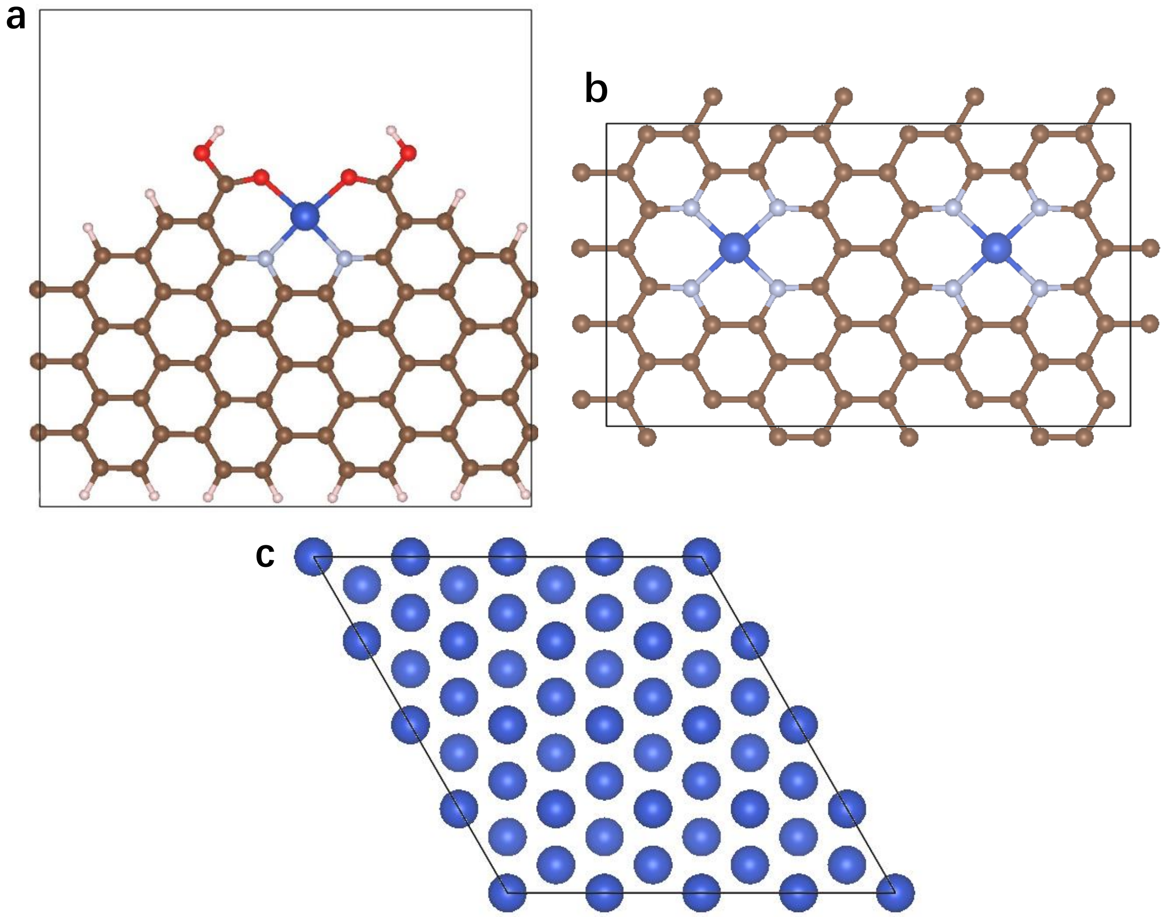

Supplementary Fig. 23. Optimized structures of (**a**) CuN_2O_2, (**b**) CuN_4, (**c**) Cu(111).

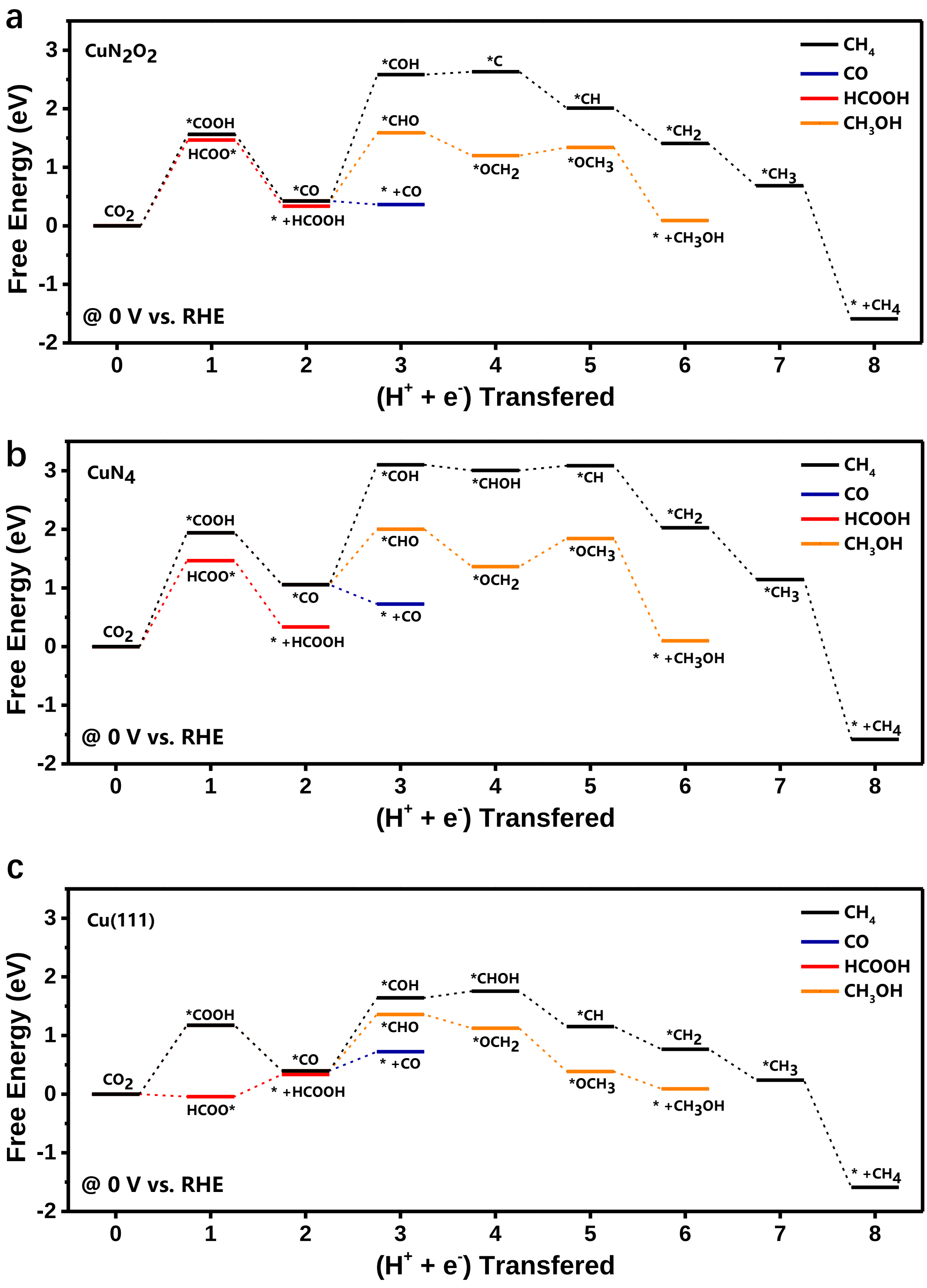

Supplementary Fig. 24. Gibbs free energy profiles for possible ECR products along the most favorable pathways for (**a**) CuN_2O_2, (**b**) CuN_4, (**c**) Cu(111).

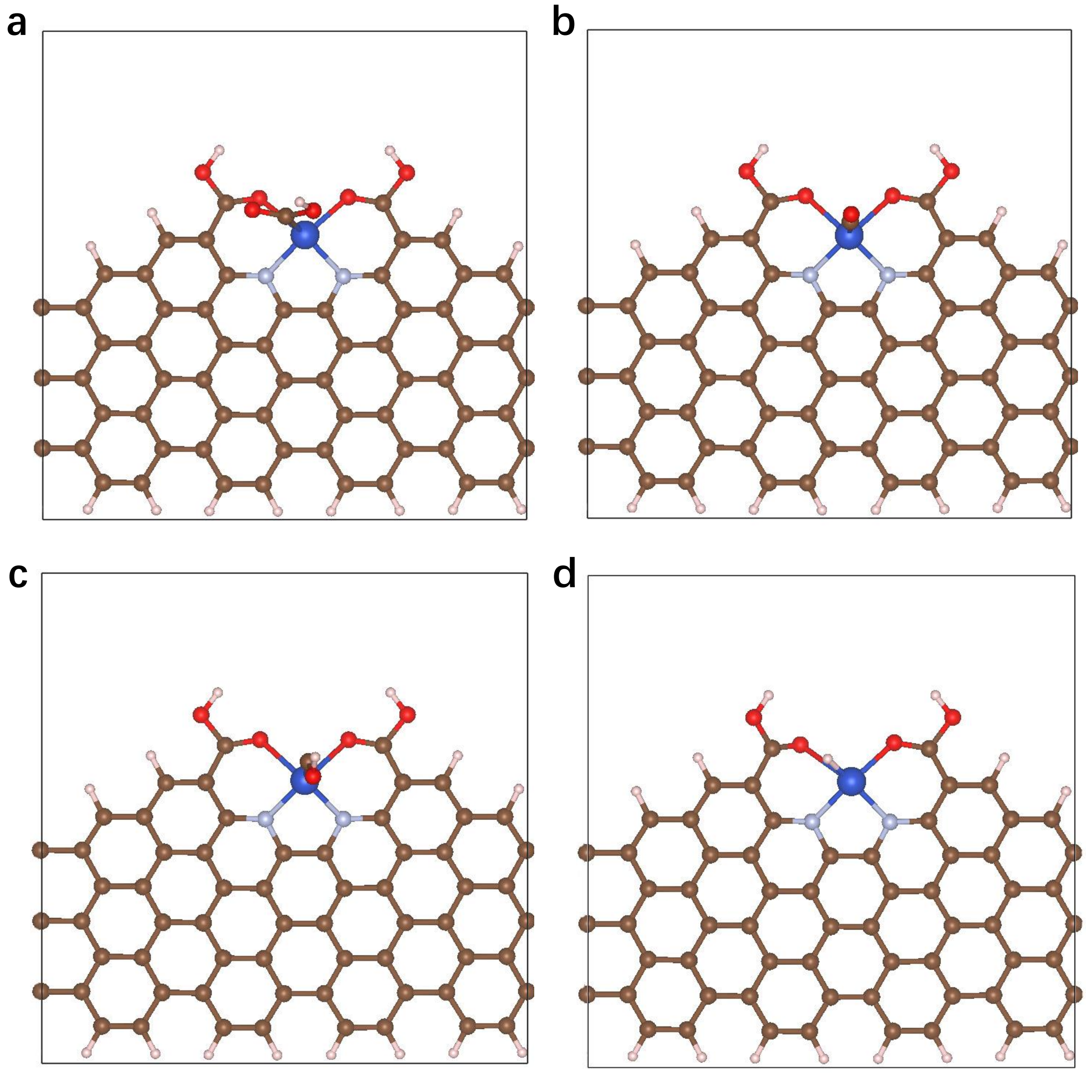

Supplementary Fig. 25. Optimized structures of (**a**) *COOH on CuN_2O_2, (**b**) *CO on CuN_2O_2, (**c**) *COH on CuN_2O_2, (**d**) *H on CuN_2O_2.

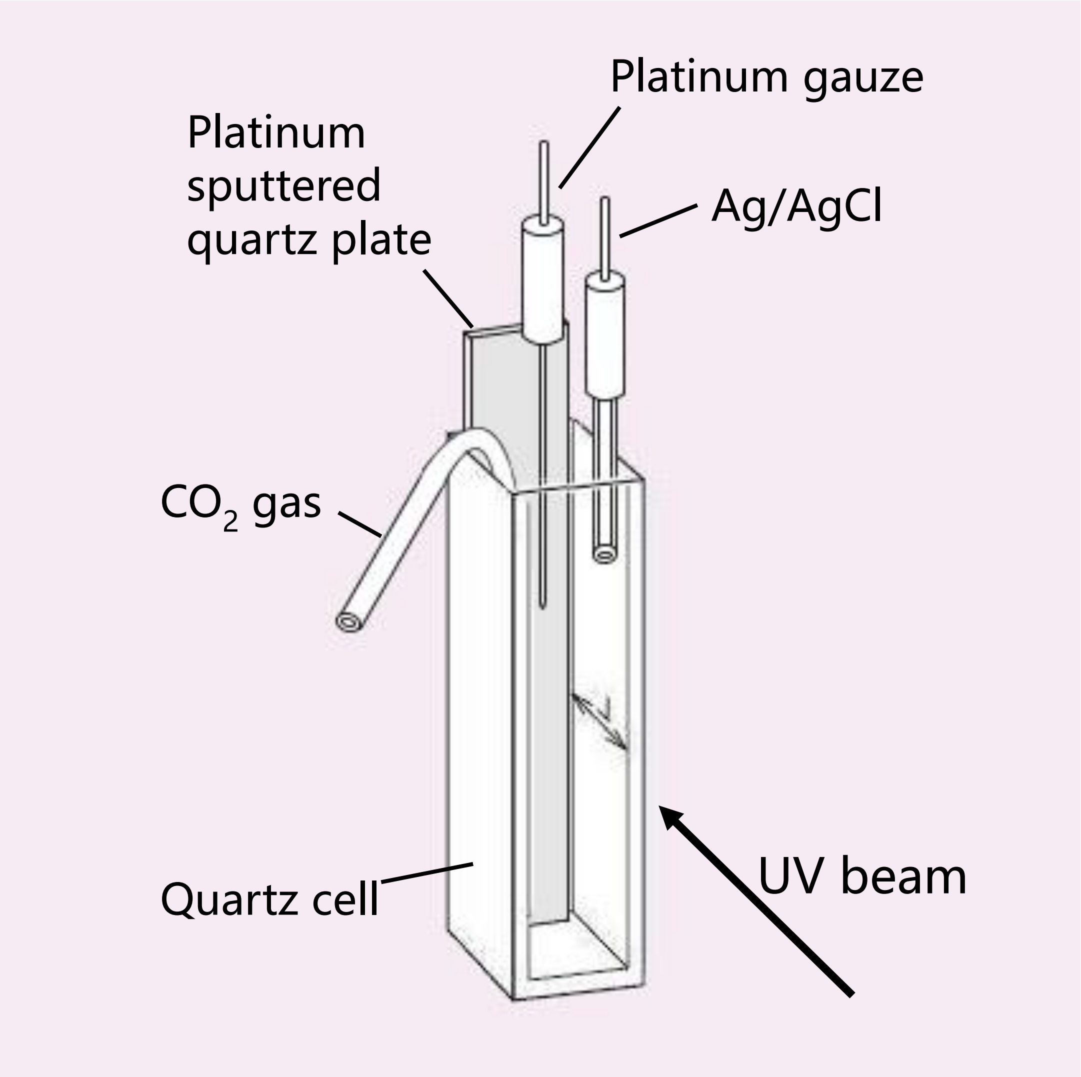

Supplementary Fig. 26. Schematic illustration of the in-situ UV–visible spectroscopy setup.

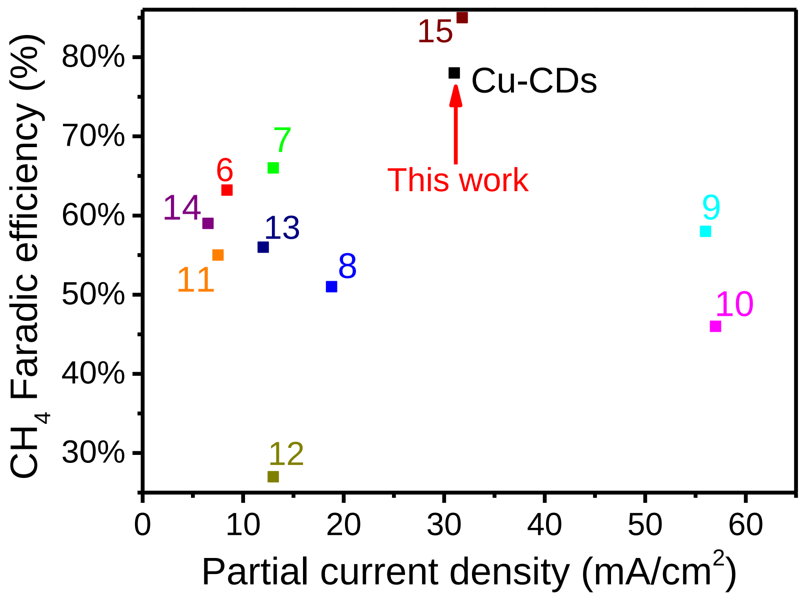

Supplementary Fig. 27. Plot of maximum CH_4 Faradaic efficiency versus CH_4 partial current density for reported catalysts, references (6-15) and details in Supplementary Table S6.

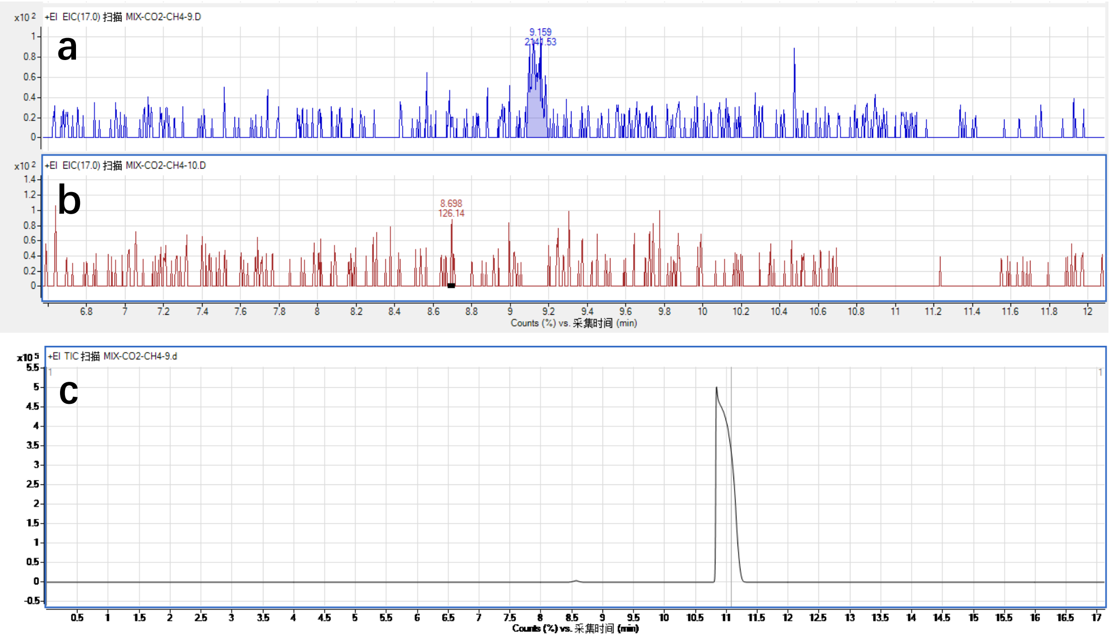

Supplementary Fig. 28. Isotope labeling results of the CH_4 generated from $^{13}CO_2$ and $^{12}CO_2$ at −1.44 V vs. RHE on Cu-CDs. Graphs of gas chromatograph mass spectrometry (GCMS) signals at m/z = 17 versus retention time (RT) of CH_4 generated from (**a**) $^{13}CO_2$ and (**b**) $^{12}CO_2$. The peaks at RT = 9.1 min are assigned to CH_4, which confirms the ^{13}C-labeled CO_2 led to the formation of the $^{13}CH_4$. (**c**) A graph of GCMS signals versus RT of CH_4 generated from $^{13}CO_2$. The peaks at RT around 11 min are assigned to $^{13}CO_2$.

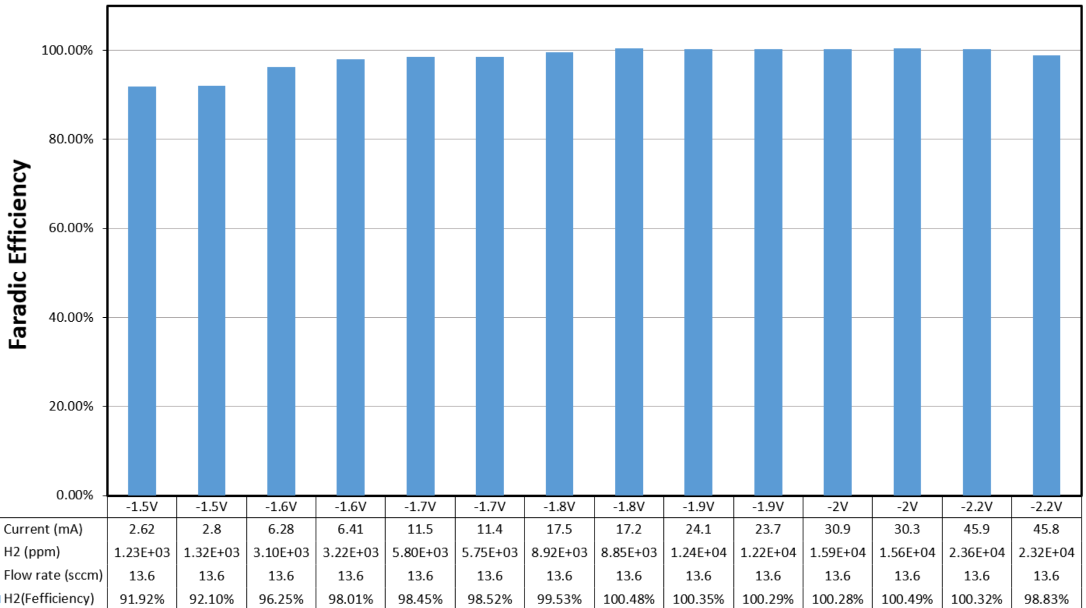

	-1.5V	-1.5V	-1.6V	-1.6V	-1.7V	-1.7V	-1.8V	-1.8V	-1.9V	-1.9V	-2V	-2V	-2.2V	-2.2V
Current (mA)	2.62	2.8	6.28	6.41	11.5	11.4	17.5	17.2	24.1	23.7	30.9	30.3	45.9	45.8
H2 (ppm)	1.23E+03	1.32E+03	3.10E+03	3.22E+03	5.80E+03	5.75E+03	8.92E+03	8.85E+03	1.24E+04	1.22E+04	1.59E+04	1.56E+04	2.36E+04	2.32E+04
Flow rate (sccm)	13.6	13.6	13.6	13.6	13.6	13.6	13.6	13.6	13.6	13.6	13.6	13.6	13.6	13.6
H2(Fefficiency)	91.92%	92.10%	96.25%	98.01%	98.45%	98.52%	99.53%	100.48%	100.35%	100.29%	100.28%	100.49%	100.32%	98.83%

Supplementary Fig. 29. Control Faradaic efficiency experiment using the N_2 as feedstock.

Supplementary Table 1. The concentrations of specific C,N configurations in Cu-CDs by XPS.

14.9 at% Nitrogen				*48.8 at% Carbon*		
Pyridinc N (at%)	***N-C (at%)***	***Pyrrolic N (at%)***	***Graphitic N (at%)***	***C=C (at%)***	***C-O (at%)***	***C=O (at%)***
10.5	2.1	1.8	0.6	39.4	3.1	6.3

Supplementary Table 2. Elemental compositions of Cu-CDs and CDs.

Sample	***Copper (at%)***	***Nitrogen (at%)(Pyridinc N (~398.8 eV))***	***Carbon (at%)***	***Oxygen (at%)***
Cu-CDs	0.28<br>(by AAS, 0.44 wt%)	14.9 (10.5)	48.8	35.9
CDs	non-detected	16.7 (7.4)	58.4	24.8

Supplementary Table 3. EXAFS fitting data for Cu center at the Cu K-edge.

Sample	***Shell***	***CN***	***R(Å)***	***σ^2*10^{-3}(Å^2)***	***ΔE_0(eV)***	***R factor***
Cu-EDTA	Cu-O/N	4.3±0.4	1.98±0.01	6.5±0.8	0.4±1.2	0.003
	Cu-C	9.9±2.3	2.86±0.02	11.1±2.5	-0.8±1.8	
Cu-CD	Cu-O/N	3.6±0.6	1.97±0.01	6.3±1.5	0.7±2.4	0.008
	Cu-C	8.3±3.0	2.86±0.03	10.3±4.1	1.6±3.0	

CN*:* coordination numbers; ***R****:* bond distance; $\boldsymbol{\sigma^2}$*:* Debye-Waller factors; $\boldsymbol{\Delta E_0}$*:* the inner potential correction; ***R factor****:* goodness of fit.

Supplementary Table 4. EXAFS fitting data with Cu–O and Cu–N two-body backscattering signals.

Sample	***Shell***	***CN***	***R(Å)***	***σ^2*10^{-3}(Å^2)***	***ΔE_0(eV)***	***R factor***
Cu-CD	Cu-N	1.7±0.3	1.91±0.02	5.3±2.4	-1.1±2.8	0.007
	Cu-O	2.0±0.5	2.01±0.02	7.2±2.3	1.7±3.4	
	Cu-C	7.2±2.6	2.87±0.03	9.3±4.0	1.6±3.0	

CN*:* coordination numbers; ***R****:* bond distance; $\boldsymbol{\sigma^2}$*:* Debye-Waller factors; $\boldsymbol{\Delta E_0}$*:* the inner potential correction; ***R factor****:* goodness of fit.

Supplementary Table 5. EXAFS fitting data with fixed coordination number of Cu–O and Cu–N.

Structure	***Shell***	***CN***	***R(Å)***	***σ^2*10^{-3}(Å^2)***	***ΔE_0(eV)***	***R factor***
CuN_3O_1	Cu-N	3*	1.93±0.01	3.5±1.4	-10.3±2.3	0.080
	Cu-O	1*	2.01±0.03	3.5±1.4	-10.3±2.3	
	Cu-C	6.0±3.7	2.92±0.07	10.4±10.1	9.2±4.8	
CuN_1O_3	Cu-N	1*	1.90±0.07	4.3±0.7	3.2±1.0	0.018
	Cu-O	3*	2.00±0.02	4.3±0.7	3.2±1.0	
	Cu-C	12.0±4.4	2.86±0.03	10.3±4.1	1.6±3.0	

CN*:* coordination numbers; ***R****:* bond distance; $\boldsymbol{\sigma^2}$*:* Debye-Waller factors; $\boldsymbol{\Delta E_0}$*:* the inner potential correction; ***R factor****:* goodness of fit. * The coordination number is fixed during the fitting. Compared to the best-fitting result (Supplementary Table 4), CuN_3O_1 and CuN_1O_3 model fits not that well with experimental spectrum of Cu-CDs (judging from *R* factor).

Supplementary Table 6. Comparison of current density and FE(CH_4) with reported catalysts.

Catalyst	*FE(CH_4)*	*Partial j_{CH_4} ($mA \cdot cm^{-2}$)*	*Other Products FE(C_2H_4 + CO + HCOOH)*	*Potential (V) vs. RHE*	*References*
Cu-CDs	78%	31	<1%	-1.44	This work
Cu_2O@Cu-MOF	63.2%	8.4	21.8%	−1.71	6
CuPc@CNTs	66%	13	9%	-1.06	7
Silver-Nanofoam	51%	18.8	23%	-1.5	8
Cu-CeO_2-4%	~58%	~ 56	20%	-1.8	9
Cu-Pd	46%	57	8%	-0.96	10
Cu NWs	55%	7.5	6%	-1.25	11
PorCu	27%	13	18%	-0.976	12
Cu RDs	56%	12	34%	-1.0	13
Polished Cu foil with I^-	59%	6.5	27%	-1.0	14
SA-Zn/MNC	85%	31.8	6%	-1.8 vs. SCE	15

Supplementary References

1. Perdew, J.P., Burke, K. & Ernzerhof, M. Generalized Gradient Approximation Made Simple. *Phys Rev Lett* **77**, 3865-3868 (1996).
2. Hammer, B., Hansen, L.B. & Nørskov, J.K. Improved adsorption energetics within density-functional theory using revised Perdew-Burke-Ernzerhof functionals. *Physical Review B* **59**, 7413-7421 (1999).
3. Blöchl, P.E. Projector augmented-wave method. *Physical Review B* **50**, 17953-17979 (1994).
4. Kresse, G. & Joubert, D. From ultrasoft pseudopotentials to the projector augmented-wave method. *Physical Review B* **59**, 1758-1775 (1999).
5. Monkhorst, H.J. & Pack, J.D. Special points for Brillouin-zone integrations. *Physical Review B* **13**, 5188-5192 (1976).
6. Tan, X. et al. Restructuring of Cu2O to Cu2O@Cu-Metal-Organic Frameworks for Selective Electrochemical Reduction of CO2. *ACS Appl Mater Interfaces* **11**, 9904-9910 (2019).
7. Weng, Z. et al. Active sites of copper-complex catalytic materials for electrochemical carbon dioxide reduction. *Nat Commun* **9**, 415 (2018).
8. Dutta, A., Morstein, C.E., Rahaman, M., Cedeño López, A. & Broekmann, P. Beyond Copper in CO2 Electrolysis: Effective Hydrocarbon Production on Silver-Nanofoam Catalysts. *ACS Catalysis* **8**, 8357-8368 (2018).
9. Wang, Y. et al. Single-Atomic Cu with Multiple Oxygen Vacancies on Ceria for Electrocatalytic CO2 Reduction to CH4. *ACS Catalysis* **8**, 7113-7119 (2018).
10. Weng, Z. et al. Self-Cleaning Catalyst Electrodes for Stabilized CO2 Reduction to Hydrocarbons. *Angew Chem Int Ed Engl* **56**, 13135-13139 (2017).
11. Li, Y. et al. Structure-Sensitive CO2 Electroreduction to Hydrocarbons on Ultrathin 5-fold Twinned Copper Nanowires. *Nano Lett* **17**, 1312-1317 (2017).
12. Weng, Z. et al. Electrochemical CO2 Reduction to Hydrocarbons on a Heterogeneous Molecular Cu Catalyst in Aqueous Solution. *J Am Chem Soc* **138**, 8076-8079 (2016).
13. Wang, Z., Yang, G., Zhang, Z., Jin, M. & Yin, Y. Selectivity on Etching: Creation of High-Energy Facets on Copper Nanocrystals for CO2 Electrochemical Reduction. *ACS Nano* **10**, 4559-4564 (2016).
14. Varela, A.S., Ju, W., Reier, T. & Strasser, P. Tuning the Catalytic Activity and Selectivity of Cu for CO2 Electroreduction in the Presence of Halides. *ACS Catalysis* **6**, 2136-2144 (2016).
15. Han, L. et al. Stable and efficient single-atom Zn catalyst for CO2 reduction to CH4. *J Am Chem Soc* (2020).
